# Accurate Genome Relative Abundance Estimation Based on Shotgun Metagenomic Reads

**DOI:** 10.1371/journal.pone.0027992

**Published:** 2011-12-06

**Authors:** Li C. Xia, Jacob A. Cram, Ting Chen, Jed A. Fuhrman, Fengzhu Sun

**Affiliations:** 1 Molecular and Computational Biology Program, Department of Biological Sciences, University of Southern California, Los Angeles, California, United States of America; 2 Marine and Environmental Biology, Department of Biological Sciences, University of Southern California, Los Angeles, California, United States of America; 3 Tsinghua National Laboratory for Information Science and Technology, Tsinghua University, Beijing, People's Republic of China; AC Camargo Cancer Hospital, Brazil

## Abstract

Accurate estimation of microbial community composition based on metagenomic sequencing data is fundamental for subsequent metagenomics analysis. Prevalent estimation methods are mainly based on directly summarizing alignment results or its variants; often result in biased and/or unstable estimates. We have developed a unified probabilistic framework (named GRAMMy) by explicitly modeling read assignment ambiguities, genome size biases and read distributions along the genomes. Maximum likelihood method is employed to compute Genome Relative Abundance of microbial communities using the Mixture Model theory (GRAMMy). GRAMMy has been demonstrated to give estimates that are accurate and robust across both simulated and real read benchmark datasets. We applied GRAMMy to a collection of 34 metagenomic read sets from four metagenomics projects and identified 99 frequent species (minimally 0.5% abundant in at least 50% of the data- sets) in the human gut samples. Our results show substantial improvements over previous studies, such as adjusting the over-estimated abundance for *Bacteroides* species for human gut samples, by providing a new reference-based strategy for metagenomic sample comparisons. GRAMMy can be used flexibly with many read assignment tools (mapping, alignment or composition-based) even with low-sensitivity mapping results from huge short-read datasets. It will be increasingly useful as an accurate and robust tool for abundance estimation with the growing size of read sets and the expanding database of reference genomes.

## Introduction

Microbial organisms are ubiquitous dwellers of the earth's biosphere whose activities shape the earth's biogeochemistry. Through pathogenesis and symbiosis, they also play important roles in the health and metabolism of macro-organisms. For example, the human body is inhabited by trillions of microbes, affecting our digestive system, immune system, and physiology [1]. Thus, the knowledge of their presence and abundance in nature is of great relevance to ecology as well as to human well-being. To study microbes in natural environments, researchers frequently apply whole genome shotgun sequencing to uncultured samples to generate genomic sequence reads reflecting the structure of microbial communities [2], [3]. Using the sequencing data, investigators try to address basic community questions such as: *who they are*, *how many they are*, and *what they do*. As a consequence of the random sampling and sequencing scheme of the shotgun metagenomics approach, the presence and abundance information of metagenomes is preserved in raw reads although some studies have shown that biases in sampling can occur, as is true for virtually all approaches [4]. However, the subsequent analysis of metagenomic data remains a challenging computational problem because of the mixed nature of metagenomes and the fact that we only sequence a small fraction of them.

Several computational methods have been developed to extract taxonomic information from metagenomic sequence reads. These existing methods can be separated into two classes: composition-based and alignment-based. In the composition-based approaches, similarity measures based on oligonucleotide composition, also known as k-mer frequencies, are used to classify metagenomic reads. For instances, TETRA, CompostBin, TACOA, and AbundanceBin are all reference-free methods and they cluster sequences with different binning strategies [5], [6], [7], [8]. PhyloPythia uses pre-trained composition-based classifiers to group sequences [9] and Phymm trains interpolated Markov model-based classifiers [10], [11]. However, none of these binning or classification approaches is designed to estimate the relative abundance of genomes for microbial communities (or the genome relative abundance (GRA)).

In the alignment-based approaches, alignment and mapping tools, such as BLAST, are commonly used to find similarity hits of the query reads to the references [12]. Some of them, such as Sort-ITEMS, use BLASTX for amino acid sequence similarity search [13]. However, we will only focus on similarity search based on nucleic acid sequence only. The MEGAN software parses BLAST results and traces back the lowest common ancestor of ambiguously assigned reads to generate a phylogenetic distribution of the reads [14]. An intuitive way of estimating GRA based on MEGAN is using the normalized read distribution along the leaves of the phylogenetic tree, leaving out the reads assigned to multiple references. However, estimation of abundance levels by this method, which discards reads with multiple origins, can be biased by many factors, including the variation of genome size [15], [16]. The latest Genome Abundance and Average Size (GAAS) tool weighs hits by their E-values and gives a direct estimation of genome relative abundance [15]. However, its accuracy and reliability are still hindered by the prevailing existence of read assignment ambiguities and the oversimplified estimation scheme.

In parallel with computational developments, significant improvements in sequencing technology have also been underway. Traditional metagenomic read sets are based on Sanger sequencing, which has an average read length at about 800 bp or above. At these lengths, taxonomic origin identification for the reads is relatively easy when the reference genomes are known. However, there was only limited availability of reference genomes as well as limited sequencing depth. Therefore, the relative abundance levels could not be accurately estimated, especially for complex communities in the past. Recent wide spread adoption of next generation sequencing (NGS) technologies in the metagenomics research community has led to the emergence of several massive, but short, read sets from Roche/454 (millions of 100–400 bp reads), Illumina/Solexa and ABI/SOLiD platforms (tens of millions of 50–100 bp reads) [17], [18].

The paradigm shift in sequencing technologies has impacted downstream analyses. Specifically, the identification of the origin of a read becomes more difficult for several reasons. First, a large number of short reads cannot be uniquely mapped to a specific location of one genome. Instead, they map to multiple locations of one or multiple genomes. These ambiguities are directly associated with the read length reduction in NGS technologies. Second, communities usually consist of many microbes with similar genomes, different only in some parts, making it indeed impossible to determine the origin of a particular short read based solely on its sequence.

Despite these difficulties, NGS read sets have brought us richer abundance information of microbial communities than traditional datasets because of the significant increase in the number of reads. Along with the increase of read set size, efforts to assemble more reference genomes are ongoing [19], [20]. In addition, new experimental techniques, such as single-cell sequencing approaches are being developed to sequence reference genomes directly from environmental samples [21], [22]. Thus, in view of the constraints of current computational tools and the fast expanding sequencing capacities, we are motivated to develop a new method for accurate and reliable GRA estimation, one that can meet the challenges of short reads and the growing number of reference genomes.

In this paper, we introduce GRAMMy, a unified Genome Relative Abundance (GRA) estimation framework using Mixture Model theory (MMy)-based modeling of shotgun metagenomic reads. Our GRAMMy framework is a reference-based method and utilizes the nucleic acid sequence similarity or composition. We first tested GRAMMy using our simulated reads as well as some synthetic communities with real reads from other studies (the FAMeS datasets) [23]. Compared to other reference-based methods, including GAAS and the abundance estimates from MEGAN, GRAMMy shows greatly improved accuracy in abundance estimations. Furthermore, with a reasonable sequencing depth, GRAMMy's estimates converge to the true abundance levels and remain stable. We then analyzed 34 real metagenomic read sets with GRAMMy, the results of which yielded interesting and new insights in biology. Finally, we packaged the GRAMMy tools as a C++ extension to Python, which can be downloaded freely from GRAMMy's homepage (http://meta.usc.edu/softs/grammy).

## Results

### The GRAMMy framework

**Figure 1:**
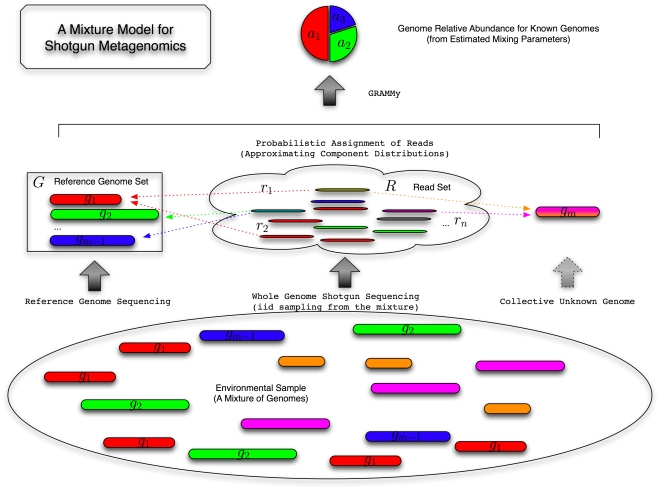
The GRAMMy model. A schematic diagram of the finite mixture model underlies the GRAMMy framework for shotgun metagenomics. In the figure, ‘iid’ stands for “independent identically distributed”.

**Figure 2:**
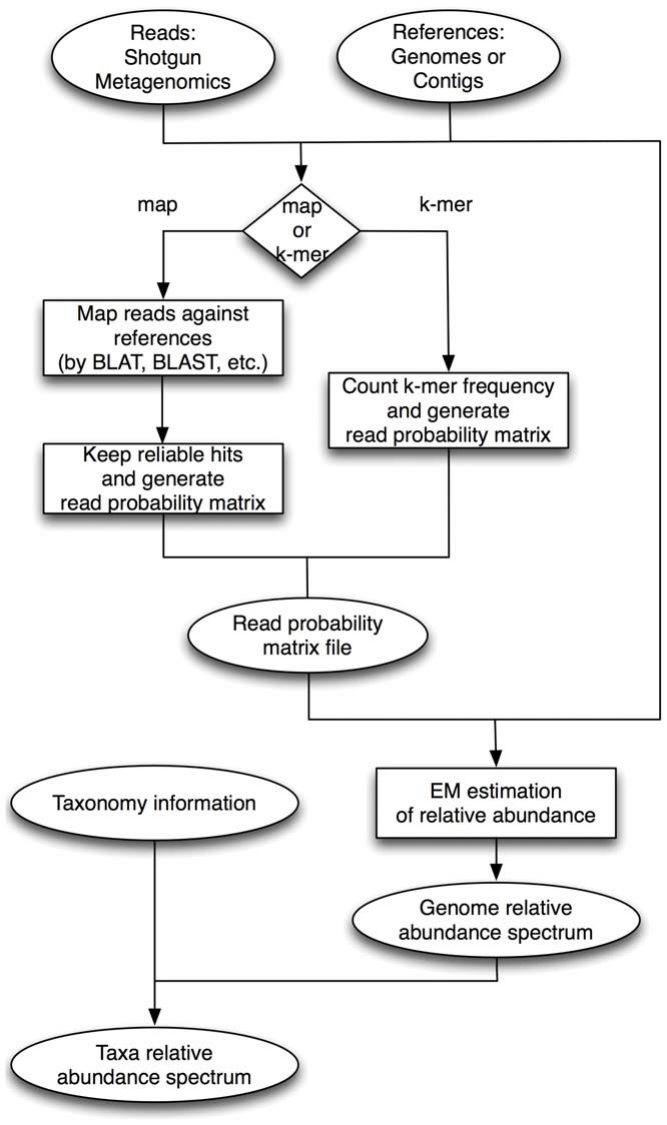
The GRAMMy flowchart. A typical flowchart of GRAMMy analysis pipeline employs ‘map’ and ‘k-mer’ assignment.

The GRAMMy framework is based on a mixture model for the short metagenomic reads and an Expectation Maximization (EM) algorithm, as outlined in the model schema and the analysis flowchart in Figures 1 and 2. GRAMMy accepts a set of shotgun reads, as well as some references (*e.g.* genomes, scaffolds or contigs) as inputs and subsequently performs the Maximum Likelihood Estimation (MLE) of the relative abundance levels. In the typical GRAMMy workflow, which is shown in Figure 2, the end user starts with the metagenomic read set and reference genome set and then chooses between mapping-based (‘map’) and k-mer composition-based (‘k-mer’) assignment options. In either option, after the assignment procedure, an intermediate matrix describing the probability that each read is assigned to one of the reference genomes is produced. This matrix, along with the read set and reference genome set, is fed forward to the EM algorithm module for estimation of the genome relative abundance levels. After the calculation, GRAMMy outputs the GRA estimates as a numerical vector, as well as the log-likelihood and standard errors for the estimates. If the taxonomy information for the input reference genomes is available, strain (genome) level GRA estimates can be combined to calculate high taxonomic level abundance, such as species and genus level estimates.

We implemented the computation-intensive core of GRAMMy in C++ with Standard Template Library (STL) for best performance and compatibility, and we integrated the typical workflow tools into a Python extension. Compared to other methods included in our study, we showed the superior accuracy and robustness of GRAMMy's estimates, as detailed in the following sections. Other choices of read assignment schema, such as NGS mapping tools and Markov Model-based read assignment [24], can also be incorporated into GRAMMy, since they produce a reasonable read assignment probability matrix. The GRAMMy package is open source, and users are able to implement other workflow variants.

### Simulated read benchmarks

We first tested GRAMMy by using a series of simulated read sets. By using read sets generated from a collection of genomes included in the FAMeS study [23], we were able to assign the true relative abundance levels and confirm the estimation accuracies by analyzing the errors between the estimates and true values. The numerical error measure RRMSE (Relative Root Mean Square Error), which computes the root mean square average of relative errors, was used to assess the accuracy and robustness of estimates. The detailed discussion of the simulation studies is provided in the Text S1 and the results are presented in Figures S1, S2, S3, S4. Figure S1 shows that all the error measures decrease to 0 as the number of reads increases. Figure S2A shows that effect of sequencing errors on the GRA estimation accuracy and it shows that sequence errors have a significant effect on short reads (<200 bp) while the effect is minimal for long reads. Figure S2B shows that missing reference genomes in the reference genome set does not significantly affect the estimation accuracy for the genomes in the reference data set even if 50% of the genomes in the community are unknown. Figure S2C shows the effect of different abundance distribution on the estimation accuracy and it shows that such an effect is not significant although we do see a slight increase in the measurement errors for communities with uneven abundance distributions compared to that for the even abundance distributions. In summary, our simulations show that the GRAMMy estimates are accurate and stable across a range of anticipated scenarios.

Interestingly, a relatively small number of short reads is sufficient to obtain an accurate estimate of relative abundance of the genomes, thus eliminating the need for an excessively ‘deep sequencing’ scheme in certain richness assessing scenarios. As shown by all panels in Figure S1, RRMSEs start to stabilize when the number of reads (*RN*) surpassed 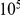, indicating the existence of a threshold for the number of reads needed to recover the community abundance structure. The trend also shows that a relatively small number of read sets could still provide substantial information for the abundance estimation, when the read assignment ambiguity is properly handled. However, the number of required reads depends on the number of organisms in the community and the distribution of relative abundances of the different organisms.

We also compared GRAMMy to other methods. With the objective of estimating the GRA of communities, we first benchmarked GRAMMy with GAAS. In addition, we included MEGAN, which produces a read profile that summarizes the number of reads assigned to their lowest common ancestors (LCA). We estimated the GRA based on MEGAN using the normalized percentages from the reads distributed on leaf taxon. In the benchmark, we used a series of simulated read sets generated from genomes randomly selected from the FAMeS study [23] (see details in Text S1). The same genomes used in read generation were also used as our reference genomes. We then used BLAT to align the reads to the reference genomes and fed the output into GRAMMy, GAAS and MEGAN. The default options of GAAS and MEGAN were used in our study. Figure S3A shows the results from the simulation read sets with read lengths (*RL*s) equal to 100 or 400 bp generated from MetaSim [25] using the ‘with sequencing errors’ option. We see that GRAMMy (‘map’) significantly outperformed GAAS, MEGAN and GRAMMy (‘k-mer’) in all settings. Among all the methods tested, GRAMMy (‘map’) is the only method with RRMSEs decreasing to zero as the number of reads increases.

To account for the poor performance of other methods, we can point to several possible reasons. For GAAS, assigning ambiguous hits based on their E-value weights is *ad hoc* and may reduce its accuracy because the E-value is only a statistical measure for the quality of the alignment. For MEGAN, its arbitrary cutoff at the top five percent hits and its non-probabilistic handling of ambiguous hits may reduce the accuracy of GRA estimates. In addition, for both MEGAN and GAAS, there is also the possibility of losing accuracy when changing from BLAST hits to BLAT hits. While it has been argued that BLAST alignment is the best way to assign reads [11], it is too computationally intensive for BLAST-aligning every read to references [18]. Instead, fast mapping tools like BLAT only keep a small number of high-similarity hits, while, at the same time, possibly reducing the accuracy of both GAAS and MEGAN. In contrast, the superior performance of GRAMMy (‘map’) shows that the probabilistic way of handling ambiguous hits could help to improve the estimation, which also gives GRAMMy an advantage over other methods when encountering incoming short read sets of very large sizes.

In conclusion, when the reference set is available, the GRAMMy framework based on mapping or alignment gave the best result for GRA estimation. Thus, the ‘map’ approach is generally the method of choice in most application settings. Only when assembled reference genomes are absent, GRAMMy (‘k-mer’) is needed as a still viable solution for GRA estimation, since at *RL* equal to 400 bp its performance is comparable to the estimates from GAAS and MEGAN. However, the k-mer composition approach has limited power to distinguish the different genomes, as the compositions of k-mer are usually heterogeneous across the genomes. In addition, there is no genome size bias correction if ‘k-mer’ method is used without prior knowledge of genome lengths.

In addition to the above methods, relative abundance estimation based on ribotype (retrieving rRNA sequences and classifying into taxonomic bins, *e.g.* 16S rRNA), protein marker (similar to ribotype method except replacing rRNA by protein marker, *e.g. rpo*B) and hit counting (counting the total number of all hits in each taxonomic bin) has been used to estimate relative abundance [26], [27], [28]. We compared the 16S-based (adapted from *Biers et al.* [28]), *rpo*B-based and BLAT hit counting estimates to GRAMMy estimates using our simulated read set. Figure S3B shows that GRAMMy outperformed all other methods in this controlled setting. All other methods show three obvious drawbacks: a persisting bias, significant variation and a strong dependence on the number of reads.

In fact, 16S rRNA and *rpo*B genes are only very small parts of genomes; therefore, even if the total number of reads is large, the reads covering these genes are barely about 1/1000 of all reads. If the total number of reads is small and there are not enough reads covering 16S rRNA genes, then the method is not viable as a result of its substantial instability. Even if the total number of reads increases, due to gene copy number and genome size variations, the estimates still do not converge to the true abundance values. Similar trends were also observed when BLAT mapping hit counts were directly normalized and used for abundance estimation. On the contrary, GRAMMy always produced much more accurate and reliable estimates.

For the estimates at different taxonomic levels, the estimation errors gradually decrease from the strain level to the kingdom level and are mostly small given a relatively large number of reads (see Figure S4).

### Artificial metagenomes with real reads

We further compared the estimates of GRAMMy with those of GAAS and MEGAN, using the third party FAMeS dataset [23]. The FAMeS data are comprised of three synthetic metagenomic read sets constructed by random sampling from real whole genome shotgun sequencing reads. These constructed read sets are labeled ‘simLC’, ‘simMC’ and ‘simHC’, according to different complexities of the communities. Each set is composed of approximately ten thousand Sanger reads from 113 microbial genomes. These artificially created metagenomes have considerably different abundance distributions, ranging from uniform-like in the ‘simLC’ set to steep power-law-like in the ‘simHC’ set, with the ‘simMC’ set in between. We ran GRAMMy (‘map’), MEGAN and GAAS on all three data sets.

The results, which are summarized in Table 1, show that the measured Relative Root Mean Square Error (RRMSE) and Average Relative Error (AVGRE) for GRAMMy (‘map’) are approximately 10–20%, while those for MEGAN-based estimates are approximately 40–50%, and those for GAAS are even larger. The benchmark further substantiates that GRAMMy (‘map’) yields the most accurate estimates for all these sets. Although the errors are not close to zero, the results are still respectable, considering that the overall sequencing depth is low in all these sets, which is, on average, less than a hundred reads per genome. The highest accuracies reachable are certainly affected by the limited number of reads and the presence of sequencing errors in these read sets. Nonetheless, recent real read sets are frequently two to three orders of magnitude larger than the FAMeS data, making accurate GRA estimation more feasible.

**Table 1:** Comparison of estimation accuracy.

	simLC		simMC		simHC	
	RRMSE	AVGRE	RRMSE	AVGRE	RRMSE	AVGRE
GRAMMy	20.0%	14.0%	25.6%	19.7%	21.6%	14.7%
MEGAN	48.6%	39.3%	50.0%	40.6%	50.2%	40.8%
GAAS	433.8%	152.5%	171.4%	111.6%	507.9%	165.8%

Table 1: Comparison of estimation accuracy. A summary of Relative Root Mean Square Error (RRMSE) and Average Relative Error (AVGRE) measured from MEGAN-based, GAAS and GRAMMy (‘map’) estimates of simLC, simMC and simHC subsets of the FAMeS data. GRAMMy (‘map’) has the lowest error rate for both error measures across all the subsets.

### Meta-analysis of human gut metagenomes

The human gastrointestinal tract harbors the largest group of human symbiotic microbes. Several shotgun metagenomics studies on these communities have been published. With more than six hundred human-related bacteria reference genomes publicly available, we are well positioned to use these datasets to illustrate the practical uses of GRAMMy. We collected ‘gut’ data from three major human gut metagenome projects including two U.S. human distal guts (∼800 bp Sanger reads, ∼100,000 reads per sample, labeled ‘hg’), 18 U.S. adult samples from twin families (∼250 bp 454 reads, ∼500,000 reads per sample, obese and lean, labeled ‘uhg’), and 13 Japanese gut samples (∼800 bp Sanger reads, ∼100,000 reads per sample, weaned or unweaned infants and adults, labeled ‘jhg’) [17], [29], [30].

For the reference set for the 33 human gut samples, we used a comprehensive collection of human gut microbes (labeled ‘HGS’), containing 388 currently available human gastrointestinal microbial genomes from multiple sources (see Table S1A). BLAT was used to assign metagenomic reads to the ‘HGS’ set according to their alignment similarities, and the overall study was labeled using the combination of the read set name, the reference genome set name, and the cut-off identity rate, such as ‘hg_HGS_90’, ‘uhg_HGS_90’, ‘jhg_HGS_90’. The results with cut-off at ‘90 percent’ identity rate are summarized in Table 2 and that from both ‘75’ and ‘90’ are provided in Tables S2, S3.

**Table 2:** Summary statistics for the metagenomic datasets.

	Mapped rate (%)			Ambiguity rate (%)			Average Genome Length (bp)		
Data (# Sets)	Med.	Min.	Max	Med.	Min.	Max.	Med.	Min.	Max.
hg_HGS(2)	46.65	43.15	50.15	31.65	30.32	32.98	2890092	2660792	3119393
jhg_HGS(13)	59.61	35.99	76.92	45.11	22.53	65.71	3745629	2268438	5657331
uhg_HGS(18)	52.35	37.49	72.51	35.90	21.65	59.81	3619072	3047940	4752910
amd_AMD(1)	45.64	46.64	45.64	1.48	1.48	1.48	2163584	2163584	2163584

Table 2: Summary statistics for the metagenomic datasets. Median (Med.), minimum (Min.) and maximum (Max.) of mapped rate, ambiguity rate and estimated average genome length for the samples: two from U.S. adult human gut (‘hg’), 13 from Japanese human gut (‘jhg’), 18 from U.S. twin families human gut (‘uhg’) and1 from acid mind drainage (‘amd’) are shown. Two reference genome sets, ‘HGS’, ‘AMD’, were used for human gut samples (‘hg’, ‘jhg’, ‘uhg’) and the acid mine drainage sample (‘amd’), respectively.

Table 2 gives the mapped rates and ambiguity rates for each data set. The mapped rate is the proportion of reads mapped at least once to the reference genomes. It can be seen from the table that 45–60%, in median, of human gut metagenomic reads were mapped to the references for all these studies. This value suggests that the reference genome set provides a good homolog resource for the human gut metagenomic reads, even though there are still several sets only showing less than 40% mapped rate.

Another quantity, ambiguity rate, is the proportion of reads that are mapped at least twice to the references. As we can see, about 21–65% of the reads were ambiguously mapped to the reference genome set across the human gut samples. While ‘uhg_HGS’ is a collection of 454 short reads, we also noticed that it has a comparable median ambiguity rate to the other two Sanger read sets. This indicates that at 250 bp, a 454 read is already as specific as a Sanger read. However, because of the ambiguities arising from the intrinsic composition of the communities, we still encountered a significant portion of reads having multiple hits regardless of their read lengths.

We estimated the relative abundances of reference genomes for these datasets and the results are summarized in Table S4. Based on these estimates, we calculated the average genome lengths for these metagenomes. The medians of genome lengths range from 2.8 Mbp to 3.7 Mbp, as shown in Table 2 and Table S3. These statistics show that the average genome lengths for the three human gut datasets are comparable. Indeed, there is no statistically significant difference in the medians of average genome length between ‘jhg’ and ‘ugh’ samples (Wilcoxon test, two-sided, P = 0.3539). The test involving ‘hg’ set is not suitable since it only contains two samples.

**Figure 3:**
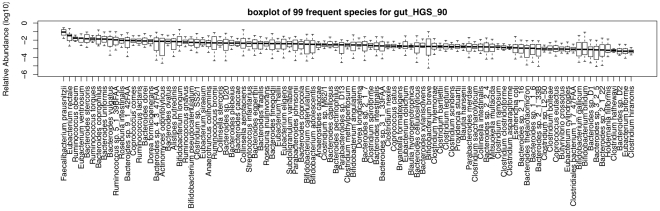
Frequent species for human gut metagenomes. The 99 species occurring in at least 50% of the 33 human gut samples with a minimum relative abundance of 0.05% were selected. ‘gut_HGS_90’ indicates that the human gut (‘gut’) read sets were mapped to the reference genome set (‘HGS’) with an identity rate cut-off at 90% (‘90’).

Next, we identified the most frequent species across all the metagenomes. In Figure 3, we show the 99 species with at least 0.05% of relative abundance in at least 50% of the metagenome samples in the order of their median relative abundance. Among the top ten most common species, there are eight from the *Firmicutes* phylum including members of *Faecalibacterium*, *Eubacterium* and *Ruminococcus* genera, and two from the *Bacteroides* genus of *Bacteroidetes* phylum. It shows the predominance of *Firmicutes* and *Bacteroidetes* in the human gastrointestinal tract. In general, these frequent species display a long-tail distribution in relative abundance levels, meaning that most species are detected across many samples, though they are not highly abundant. We also found that the abundance levels of some species are highly variable, while most others are relatively constant (see the quantile boxes and outliers in Figure 3). In choosing the minimum occurrence rate and minimum abundance threshold for a typical human gut read set (∼100,000 reads, 800 bp), the 0.05% of relative abundance roughly corresponded to a sequencing size of 40 Kbp from the genome. This size was 25-fold more than the size coverage per genome using 16S RNA sequencing according to *Qin et al.* [18]. We used a different identity rate cut-off (75%) for parsing BLAT hits and similar frequent species results were obtained. They are shown in Figure S5.

We compared our results to the 75 non-redundant, frequent species identified in a recent study [18]. Although we used different datasets and methodologies, our study shows comparable results. For example, between the two identified sets, five of the top ten common species are shared and so are eleven of the top twenty. The criteria they used (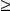
1% genome coverage and 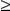
50% presence), if converted, roughly correspond to 0.05% in minimum relative abundance levels in our study.

However, we had some improvements over their results. They used a smaller (195) reference genome set and did not consider the genome size bias and the ambiguous hits. Consequently, their result might have missed some of the top frequent species and misplaced some species into the top rankings. In fact, the *Bacteroides* species, with genome lengths ranging from 5 Mbp to 8 Mbp, well above the median average genome lengths of human gut samples, are constantly ranked higher in their ranking. In our results, however, this bias is corrected, and the rankings are accordingly lowered, with some of their top 20 ranked *Bacteroides* species dropping out of the top 40.

**Figure 4:**
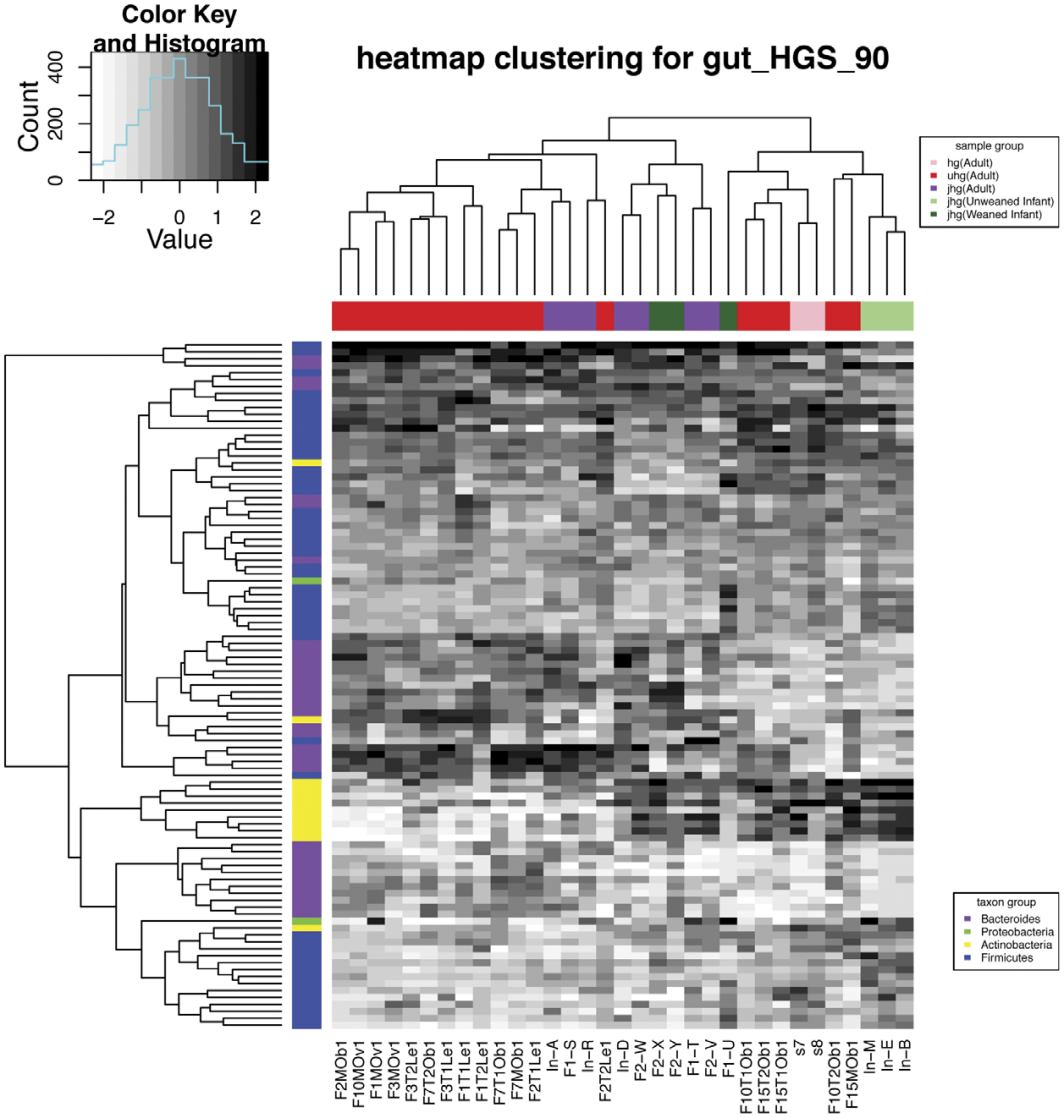
Heatmap biclustering of human gut metagenomes. ‘gut_HGS_90’ indicates that the human gut (‘gut’) read sets were mapped to the reference genome set (‘HGS’) with an identity rate cut-off at 90% (‘90’). The bottom labels indicate human gut samples. The top right legend shows the color-coding for columns indicating the sample age category and dataset origin. The bottom right legend shows color-coding for rows indicating the top 4 most abundant phyla in human gut. The relative abundance for each sample is normalized by a rank transformation.

Next, we used the GRA estimates for frequent species as the basis for hierarchically clustering all the human gut samples, as shown in Figure 4. It can be seen that most of the frequent species belong to *Firmicutes*, *Bacteroides* and *Actinobacteria* (see column color-coding). We also see that the unweaned infants (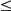
6 months) are all grouped closely together (see row color-coding), possibly indicating their distinct gut microbial communities in comparison to that for the weaned infant and adult samples. This phenomenon was noticed in the original paper [30], and our results further strengthened their claim by incorporating data from more human gut metagenomics studies. A close look at the top 20 most abundant strains revealed that the unweaned infants' community profiles were dominated by only a few strains from *Actinobacteria*. The lack of diversity of infant gastrointestinal tract has also been reported in other studies, for example, see *Vaishampayan et al.* [31]. The pattern might be related to the microbial colonization process of infant gastrointestinal tract; however, no clear explanation for this interesting phenomenon is available to date.

On the other hand, there is no clear-cut evidence showing that samples from the same dataset or Body Mass Index (BMI) category are grouped together, even though there is such a trend. Note that the clustering results depend on the criterion of identifying frequent species. These species were chosen as a trade-off between the number of frequent species required for resolution power and the number that would risk including too many unreliable estimates from less abundant species. The parameters we had chosen were based on *Qin et al*. [18]. We did the same analysis with a different identity rate cut-off (75%) for BLAT hits and two different minimum relative abundance thresholds (0.01% and 0.1%) for frequent species selection. Similar results were obtained. They are shown in Figures S6.

### The acid mine drainage data set

In samples from other environments where reference genomes are not well characterized, such as soil, ocean and some extreme environments, assemblages like contigs and draft genomes from the sample itself can be used in addition to available known genomes. Acid mine drainage sites are extreme environments where only a few species of specially adapted microbes can survive. We downloaded the raw read set (labeled ‘amd’), which contains 103,462 Sanger shotgun reads (∼750 bp) from one environmental sample of a biofilm [3]. The genome sequences of coexisting species were partially assembled using the metagenomic reads, among which are two dominant ones: *Ferroplasma sp. Type II* and *Leptospirillum sp. Group II 5-way CG*. The genome assemblages are in the draft state, but we roughly know their genome sizes [3]. To study the community structure, we constructed an acid mine drainage reference genome set (‘AMD’) using the two draft genomes and other currently available bacterial genomes of acid mine habitats (Table S1B). We mapped the read set ‘amd’ to this reference genome set and subsequently labeled the result ‘amd_AMD’.

**Figure 5:**
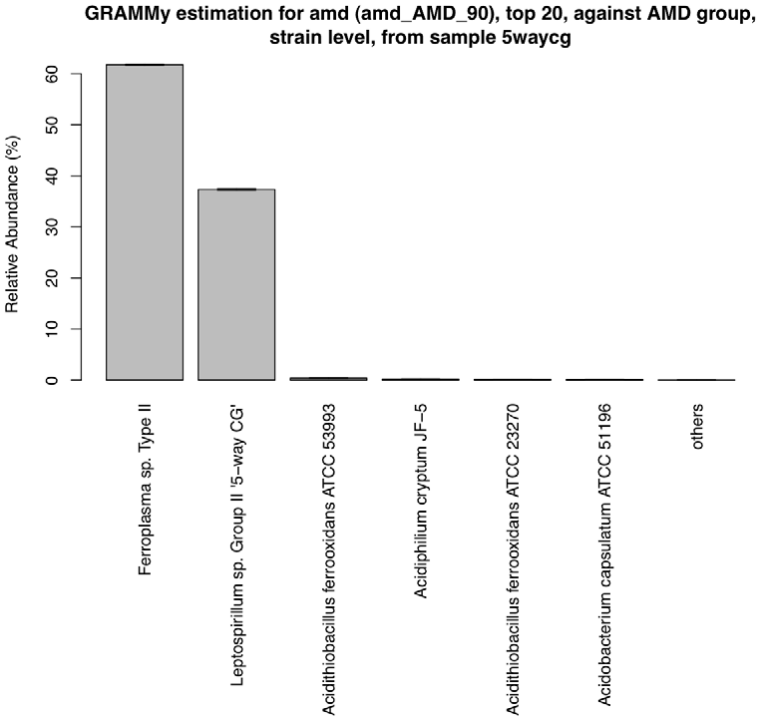
GRAMMy estimates of GRAs for the acid mine drainage data. Estimated relative abundance for each strain is shown as a percentage. The first two strains dominate the sample.

Out of the reads mapped to the references, only a slight portion of them (∼2%) had multiple hits (Table 2). We then estimated the GRA for the acid mine drainage community using GRAMMy. Figure 5 shows the relative abundance of the six strains we included in the ‘AMD’ reference. It confirms that the community is dominated by the two draft genomes (98% in total relative abundance) with only marginal fraction of the other acid mine strains. The dominance of the two strains is consistent with the results from the genomic study in the original work, even though their fluorescence in-situ hybridization (FISH) result only reveals the dominance of *Leptospirillum sp. Group II* species [3].

## Discussion

We have developed the GRAMMy framework for estimating genome relative abundance with shotgun metagenomic reads. It has three unique features. First, it is unique in providing a rigorous probabilistic framework for estimating Genome Relative Abundance (GRA). The estimation can be easily extended to higher taxonomic levels by simply adding up the relative abundance of genomes affiliated with the specific higher-level taxon while maintaining the accuracy, since the estimated GRA is already properly normalized and corrected for genome size bias.

**Figure 6:**
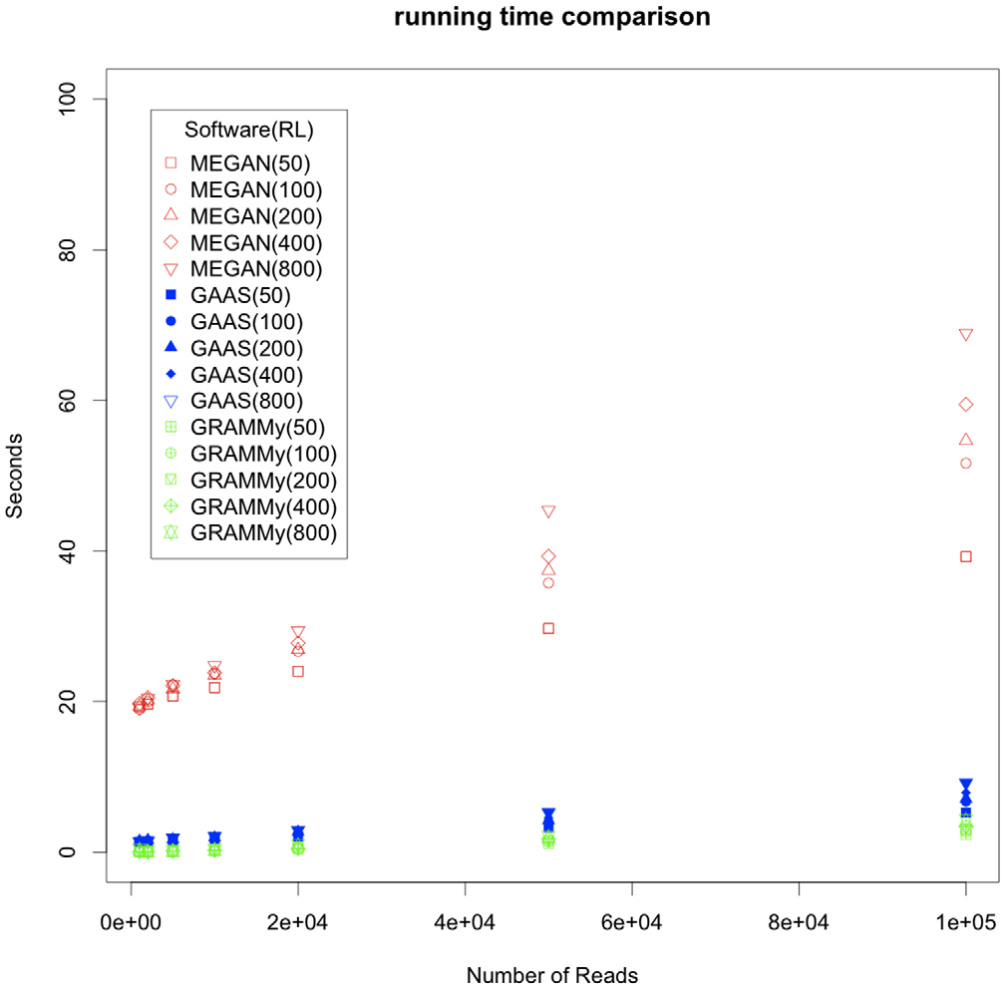
Running time comparison. GRAMMy is the fastest in all cases as compared to MEGAN and GAAS in processing time. The BLAT mapping time is excluded for all compared tools.

Second, GRAMMy provides users with a wide choice of mapping and alignment tools. Its ability to use the results from linear time NGS mapping tools helps to reduce the computation burden for analyzing current massive metagenomic read sets. The GRAMMy program currently supports tabular BLAST formats, however, the mapping results from other popular mapping tools, such as MAQ, Bowtie and PerM [32], [33], [34], can be easily adapted to the GRAMMy framework. The algorithm is also linear in time and space with the input data size and the current implementation is much faster than MEGAN and GAAS in handling large read sets, processing one million of reads in seconds (see Figure 6, the BLAT mapping time is excluded for all compared tools). In addition, GRAMMy is memory efficient and we have not encountered problems in processing read number in the order of millions with hundreds of microbial genomes with our 12GB nodes. However, if memory bottleneck is reached, we can always divides the reads into sub-samples and use GRAMMy in a bootstrap fashion, because a certain number of reads can already provide substantial amount of abundance information as indicated by our simulations.

Third, the method is especially suitable for short read datasets due to its better handling of read assignment ambiguities. In typical cases of a short read set, there are 10% to 40% of reads having assignment ambiguities [14]. The source of assignment ambiguity can be sequencing errors, genetic variations, horizontal gene transfers or closely related genomes. By taking into account the information from the ambiguously assigned part of the read set, our study showed that we can improve the genome abundance estimation for metagenomic data.

In applying the GRAMMy framework to the real metagenomic datasets, we used two different identity rate cut-offs: 75% and 90%. While the results from 90% were shown, we also kept the 75% results in the supplementary files. Lowering the thresholds will certainly increase the mapped rate as well as the ambiguity rate, as shown in Table S2. However, in our analysis of human gut metagenomes, the average genome size estimates and abundance estimates were not significantly changed by using different cut-offs, as shown in Tables S3 and S4. Still, in other applications, researchers have to trade off between ambiguity rate and mapped rate to obtain reasonable GRA estimates for their data.

There is also the practical question of how many genomes to be included as reference. This, however, is always the choice of users. As long as the read-to-genome associations found by mapping tools are reliable and the coverage rate is high (as in our simulations), GRAMMy can reliably estimate low abundance levels and the concern of over-fitting can be alleviated. In real data, the estimation accuracy of the GRA of the low-abundance genomes depends on the number of reads mapped to each genome and the reliability of mappings. The estimated variance of the GRAs can give some ideas about the accuracy of the estimates.

In summary, with the experimental side of shotgun metagenomics accelerating its pace, the GRAMMy method we proposed has the potential to produce more accurate taxonomic abundance estimations for downstream computational analyses.

## Materials and Methods

### A finite mixture model

We developed a finite mixture model for the GRAMMy framework. Following *Angly et al.* we used genome relative abundance (GRA) as the relative abundance measure of mostly unicellular microbial organisms [15]. We describe the sampling and sequencing procedure as follows: First, randomly choose a genome 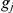 with probability 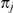 proportional to 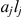, where 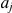 is the abundance and 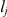 is the genome length. Second, randomly generate a read 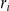 from it. Without loss of generality, we further assume that for the given genome 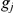 we can reasonably approximate the generation of shotgun reads by some component distribution 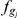 such that the probability of generating a read 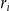 from 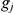 is 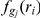. With a reasonable assumption of independence between the two sampling steps, the whole procedure is probabilistically equivalent to sampling from a mixture distribution 
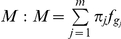
, with the mixing parameters denoted by 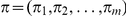, 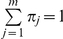 and the component distributions denoted by 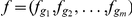, where *m* is the number of genomes. Subsequently, each read set, denoted by 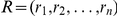, can be regarded as a realized independent, identically distributed (iid) sample of size 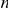 from the mixture 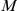. The relative abundance of known genomes is exactly a transformation of the mixing parameters 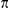, which can be estimated based on the read set 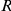. A schematic view of the finite mixture model is shown in Figure 1. With the component distributions properly set up, we can find the maximum likelihood estimate (MLE) of the mixing parameters.

In many studies, our knowledge of the genomes present in the community is limited. Under these circumstances, we can define the mixture with the first 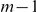 components for known genomes and the last 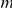-th component for the collective of unknown genomes. Note that for the 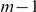 known components, we suppose that their genome sequences 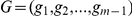 and genome sizes 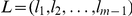 are known. Therefore, the GRA for known genomes 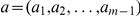 is the normalized abundance, where the relative abundance for the 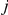-th known genome is 
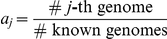
, where 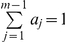. In the biological setting, we want to estimate vector 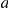, which is a measure of organism relative abundance. In the transformed mixture problem, 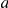 is related to the mixing parameters 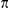 by:
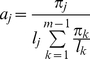
(1)or the inverse:
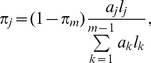
(2)for 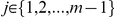. The number of sampled reads is both proportional to the genome relative abundance and the length. Because the two factors are confounded, the missing knowledge of the genome length 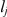 prohibits the estimation of 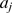 from the data. Since the effective genome length 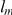 for the unknown genomes is not available, we cannot estimate the relative abundance of the unknown component. However, the relative abundance of known genomes can still be estimated using our procedures.

### Estimation of GRA using Expectation Maximization (EM) algorithm

To estimate the mixing parameters, we adopted the EM algorithm to calculate the maximum likelihood estimate (MLE). In the EM framework, we assume a ‘missing’ data matrix 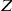, in which each entry 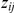 is a random variable indicating whether 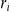 is from 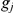 or not. Then we can solve for the parameters by iteratively estimating 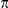 and 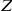 using Algorithm 1 (see supporting Methods). Note that a variable with superscript 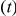 stands for its value at the 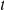-th iteration, e.g., 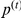 is the estimate of 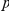 at the 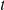-th step. The EM at the 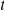-th iteration is:

- **E-step**

Assuming 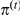 known, 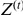 can be updated by the corresponding posterior probabilities:
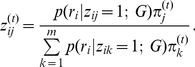
(3)

- **M-step**

Assuming 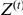 known, the new mixing parameters 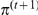 are updated by:
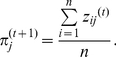
(4)When the MLE of 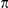 is found, using Equation (1), the MLE of 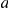 can be calculated, thereby solving the original problem.

The space complexity of the EM algorithm is 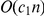 and the time complexity of the EM algorithm is 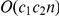, where 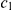 the average number of associated genomes for one read and 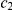 is the time cost related to the convergence criteria for EM. Since 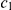 and 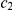 are both constants not related to *n*, the algorithm is linear in space and time complexity with the read number 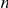. Further, the concavity of the log-likelihood function can be shown and the EM algorithm is guaranteed to converge to global maximum (see Text S1).

### Read probability approximations

The probability 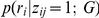 is assessed based on 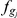. Ideally, it is the probability that 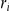 is generated when read being uniformly sampled from genome 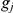. Let 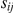 be the number of copies of read 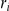 in 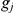. Then the probability is approximated by:
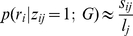
(5)However, due to sequencing errors and natural genetic variations, the 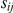
's are not readily observable. When the mapping or alignment results from BLAST, BLAT, or other mapping tools are available, the number of high quality hits of 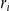 on 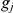 can effectively be used as 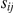
's. To keep only these reliable and statistically significant hits, raw hits are filtered by E-value, alignment length and identity rate. We refer to the finite mixture model with the read probability from mapping and alignment results as ‘map’ in the remainder of the paper.

An alternative way to assess the read probabilities is by using k-mer composition. For the *j*-th genome, we calculate the fraction of a k-word *w* by 
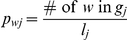
, the normalized frequency of the word 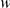 in genome 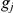. For a read 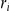, we define pseudo-likelihood for 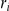 by:
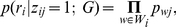
(6)where 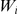 is the set of words formed by sliding windows of size *k* along 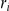. This probabilistic assignment captures the overall similarity between reads and genomes, an idea adopted in other composition-based studies such as in *Sandberg et al.* [35]. It is especially useful when a large number of reads do not have reliable hits with reference genomes. We will refer to the finite mixture model with the read probability from the multinomial k-mer composition as ‘k-mer’ in the remainder of the paper.

### Standard errors for GRA estimates

We also derived the asymptotic covariance matrix for the mixing parameters 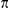 using the asymptotic theory for MLE estimates. Because there are 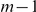 independent parameters in 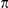, we can choose them as 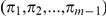 and denote by 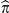. Further, let 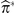 and 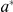 be the MLE estimates for 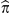 and its corresponding GRA, respectively. Then, the asymptotic standard error for 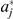 is approximately:
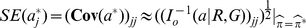
(7)for 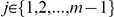, where 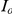 is the observed information matrix.

If only a small number (as compared to number of parameters) of reads are mapped, the conditions for the asymptotic to hold cannot be satisfied. We can alternatively use the bootstrap covariance estimator for the standard error of MLE:
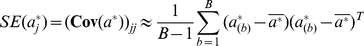
(8)for 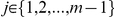, where 
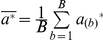
 is the bootstrap mean estimator.

### Numerical error measures

We use the following measures to evaluate the accuracy of the GRA estimate. Let the true GRA be 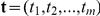 and its estimate 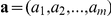. The first measure is the commonly used root mean square version of relative error 
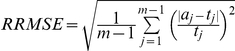
 [36]. We also included three other error measures: 
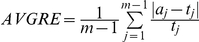
 (the average relative error), 
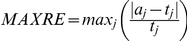
 (the maximum relative error), and 
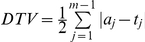
 (the *Total Variation Distance* [37]), which are all commonly used to evaluate the accuracy of an estimate.

### Real read sets and reference genome sets

In preparing the real read sets, we downloaded the FAMeS data from JGI (http://FAMeS.jgi-psf.org), the ‘hg’ data from TraceDB (ftp://ftp.ncbi.nih.gov/pub/TraceDB/, NCBI project id: 16729), the ‘uhg’ data from Sequence Read Archive (http://www.ncbi.nlm.nih.gov/Traces/sra/, NCBI project id: 32089), the ‘jhg’ data from BGI (http://gutmeta.genomics.org.cn/) [30] and the ‘amd’ data from TraceDB (NCBI project id: 13696).

In preparing the reference genome sets, we downloaded currently available complete and draft bacteria genomes from the NCBI Refseq (http://ftp.ncbi.nih.gov/refseq), MetaHit (http://www.metahit.eu/), HMCJ (http://metagenome.jp), WUSTL Gordon Lab (http://genome.wustl.edu/) and JGI (http://genome.jgi-psf.org/). We manually curated genomes to remove redundancy and organized them into a NCBI Taxonomy (http://www.ncbi.nlm.nih.gov/Taxonomy) database. We used the genome information available from IMG/M (http://img.jgi.doe.gov), IMG/HMP (http://www.hmpdacc-resources.org/cgi-bin/img_hmp) and GOLD (http://www.genomesonline.org) to group them by habitats [38], [39]. Finally, we obtained 388 human gastrointestinal tract genomes for a human gut reference genome set (‘HGS’).

### Read filtering and assignment procedures

In the ‘map’ read probability backend, we used BLAT to map reads to reference genomes. We prefer BLAT to BLAST, as BLAT is tens of times faster in handling low-sensitivity similarity search for massive number of sequences than BLAST. Since we only kept alignment results with identity rate greater than 90%, the BLAT result should not differ much from what if BLAST was used. For the human gut and simulated data, we used similar filtering methods as by *Turnbaugh et al.* [17], [40] (E-value ≤0.0001, aligned length more than 75% of its *RL* and identity ≥90%). In the ‘k-mer’ read-probability backend, we used k-mer length k = 6. For GAAS and MEGAN, we used the same mapping results from BLAT, as a common starting point. We used GAAS's default filtering options (E-value≤0.0001, aligned length more than 80% of its *RL*, and identity ≥80%), as well as MEGAN's default options (min-score = 35 for *RL* equal to 100 bp and min-score = 50 for *RL* equal to 400 bp; top percent = 5%, min support = 2), for comparisons.

In evaluating the ribotype and protein marker based method, we used the *E.coli* 16S rRNA *rrs*E and ribosome protein *rpo*B genes to retrieve homolog sequences from the simulated reads, which were then filtered by options (E-value
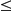
0.0001, aligned length more than 75% of its *RL* and identity 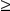
90%), according to [28]. Our validations have shown that variations of these parameters within a reasonable range had little effect on the results.

### Higher level taxonomic statistics

Many downstream analyses can be carried out based on GRAMMy's estimates. For example, the average genome length 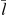 is readily obtainable:
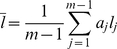
(9)Subsequently, we can test the statistical significance of the median average genome length difference between two sample groups by Wilcoxon test (wilcox.test in R).

Since genome size bias has already been corrected, we can use GRAMMy estimates to calculate the relative abundance of a higher-level taxon by simple addition. For this purpose, we used the NCBI Taxonomy, which has the taxonomic assignments for all reference genomes we used here. To illustrate, for a specific taxonomic level 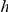, the relative abundance of a 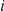-th specific taxon 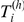 is:
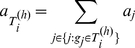
(10)and
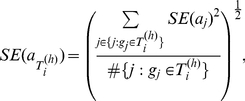
(11)where 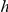 can be any one of the seven hierarchical levels in the taxonomy, from species to kingdom.

### Hierarchical biclustering

It is possible to use GRAMMy estimates for clustering analysis and statistical hypothesis testing. We clustered the samples based on the pairwise similarities (correlations) of their relative abundance distribution. Because of the long-tailed shape of the distribution, the signal-to-noise ratio is low for these less abundant genomes. Therefore, using the thresholds .05% for the minimum abundance and 50% for the minimum occurrence [18], we selected the estimates for these more abundant genomes (which are more reliable for clustering). We used rank transformation, which normalizes GRAs by taking their ranks and applying score transformation and R function heatmap for hierarchical clustering.

## Supporting Information

Figure S1 **The convergence of GRAMMy.** The estimation errors, as measured by different numerical methods: (A) Relative Root Mean Square Error (RRMSE) in percentage versus Read Number (RN) for different read lengths (RL). (B) Relative Root Mean Square Error (RRMSE), Average Relative Error (AVGRE), Maximum Relative Error (MAXRE), and Distance of Total Variation (DTV) versus Read Number for read length equal 100 bp. GRAMMy (‘map’) was used.
(TIFF)

Figure S2 **Simulated read set benchmarks.** Effects of different perturbations on GRAMMy's estimation: (A) Effects of sequencing errors: results from ‘with sequencing error’ and ‘without sequencing error’ read sets are labeled as ‘w. Seq Err’ and ‘wo. Seq Err’, respectively. (B) Effects of unknown genomes: results from estimation ‘with unknown genomes’ and ‘without unknown genomes’ read sets are labeled as ‘w. Unknowns’ and ‘wo. Unknowns’, respectively. (C) Effects of different genome relative abundance distributions: results from more concentrated abundance distribution and less concentrated read sets are labeled as ‘steep’ and ‘flat’, respectively. Relative Root Mean Square Error (RRMSE) as a percentage is plotted against Read Number. GRAMMy (‘map’) was used.
(TIFF)

Figure S3 **Performance comparison of different methods.** The performance comparisons for different estimation methods: (A) MEGAN-based (‘MEGAN’), GAAS (‘GAAS’) and GRAMMy (‘map’ and ‘k-mer’) on simulated read sets with sequencing errors at read length 100 bp and 400 bp. (B) 16S-based (‘16S’), BLAT hit counting (‘BLAT’), *rpo*B-based and GRAMMy (‘map’). Relative Root Mean Square Error (RRMSE) as a percentage is plotted against Read Number (RN).
(TIFF)

Figure S4 **Estimation errors at different taxonomic levels.** Average Relative Error (AVGRE) as a percentage is plotted against taxonomic level. The errors gradually decrease from strains to kingdom taxonomic levels.
(TIFF)

Figure S5 **Frequent species for the human gut metagenomes.** The 99 species occurring in at least 50% of the 33 human gut samples with a minimum relative abundance of 0.05% were selected. ‘gut_HGS_75’ indicates that the human gut (‘gut’) read sets were mapped to the reference genome set (‘HGS’) with an identity rate cut-off at 75% (‘75’).
(TIFF)

Figure S6 **Heatmap biclustering of the human gut metagenomes.** ‘gut_HGS_90’ indicates that the human gut (‘gut’) read sets were mapped to the reference genome set (‘HGS’) with an identity rate cut-off at 90% (‘90’), while ‘gut_HGS_75’ indicates cut-off at 75%(‘75’). The bottom labels indicate human gut samples. The top right legend shows the color-coding for columns indicating the sample age category and dataset origin. The bottom right legend shows color-coding for rows indicating the top 4 most abundant phyla in human gut. (A) Heatmap clustering of the ‘gut’ samples, with strains of abundance >0.05% in at least 50% of samples selected at 75% identity rate cut-off. (B) Heatmap clustering of the ‘gut’ samples, with strains of abundance >0.01% in at least 50% of samples selected at 90% identity rate cut-off. (C) Heatmap clustering of the ‘gut’ samples, with strains of abundance >0.1% in at least 50% of samples selected at 90% identity rate cut-off.
(TIFF)

Table S1 **Reference genome sets.** Columns are NCBI taxon ID (‘NCBI Taxon ID’), organism name (‘Name’), genome project status (‘Status’: ‘D’ for draft and ‘F’ for finished), and data source of genome sequences (‘Source’). (A) ‘HGS’ reference genome set. (B) ‘AMD’ reference genome set.
(XLS)

Table S2 **Mapping rate statistics.** Columns are read set name (‘Set Name’), total number of reads (‘Total Reads’), number of mapped reads (‘Mapped Reads’), proportion of mapped reads (‘Mapped rate’), number of ambiguous reads(‘Ambiguous Reads’), proportion of ambiguous reads (‘Ambiguous rate’). ‘xxx_yyy_zzz’ abbreviation is as specified in Methods, where ‘xxx’ is the read set, ‘yyy’ is the reference genome set and ‘zzz’ is the cut-off for identity rate. (A) ‘hg_HGS_90’. (B) ‘jhg_HGS_90’. (C) ‘jhg_HGS_90’. (D) ‘amd_AMD_90’. (E) ‘hg_HGS_75’. (F) ‘jhg_HGS_75’. (G) ‘uhg_HGS_75’. (H) ‘amd_AMD_75’. (I) Median (‘Median’), minimum (‘Min’) and maximum (‘Max’) summary of mapped ratios in panels (A–H). (J) Median (‘Median’), minimum (‘Min’) and maximum (‘Max’) summary of ambiguous ratios in panels (A–H).
(XLS)

Table S3 **Average genome length.** Average genome length estimates from GRAMMy. ‘xxx_yyy_zzz’ abbreviation is as specified in Methods, where ‘xxx’ is the read set, ‘yyy’ is the reference genome set and ‘zzz’ is the cut-off for identity rate. Median (‘Median’), minimum (‘Min’) and maximum (‘Max’) summary of GRAMMy estimated average genome length.
(XLS)

Table S4 **GRAMMy estimates of GRAs for the human gut samples.** Each row represents a data set and each column represents a species. The excel file name is abbreviated as ‘xxx_yyy_zzz’ , where ‘xxx’ is the read set, ‘yyy’ is the reference genome set, and ‘zzz’ is the cut-off for identity rate. A) ‘hg_HGS_90’. (B) ‘jhg_HGS_90’. (C) ‘jhg_HGS_90’. (D) ‘hg_HGS_75’. (E) ‘jhg_HGS_75’. (F) ‘uhg_HGS_75’.
(XLS)

Text S1 **Simulation details and technical derivations.**
(PDF)
