# Supplementary figures and images for: Accurate Genome Relative Abundance Estimation Based on Shotgun Metagenomic Reads
Source: PLoS One. 2011 Dec 6;6(12):e27992. doi: 10.1371/journal.pone.0027992 (PMC3232206; doi:10.1371/journal.pone.0027992)

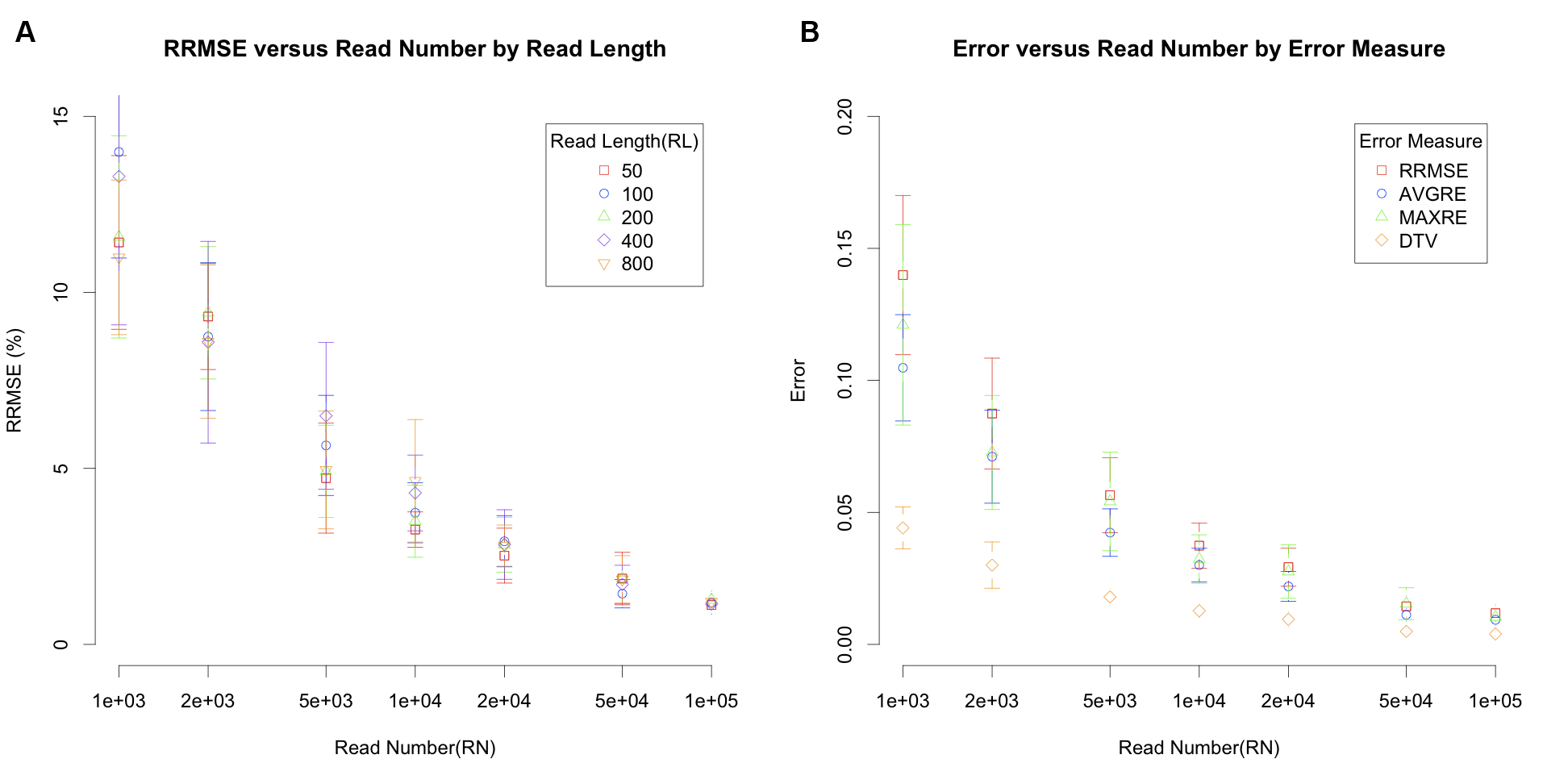

Supplement: Figure S1 — The convergence of GRAMMy. The estimation errors, as measured by different numerical methods: (A) Relative Root Mean Square Error (RRMSE) in percentage versus Read Number (RN) for different read lengths (RL). (B) Relative Root Mean Square Error (RRMSE), Average Relative Error (AVGRE), Maximum Relative Error (MAXRE), and Distance of Total Variation (DTV) versus Read Number for read length equal 100 bp. GRAMMy (‘map’) was used. (TIFF) [file pone.0027992.s001.tif]

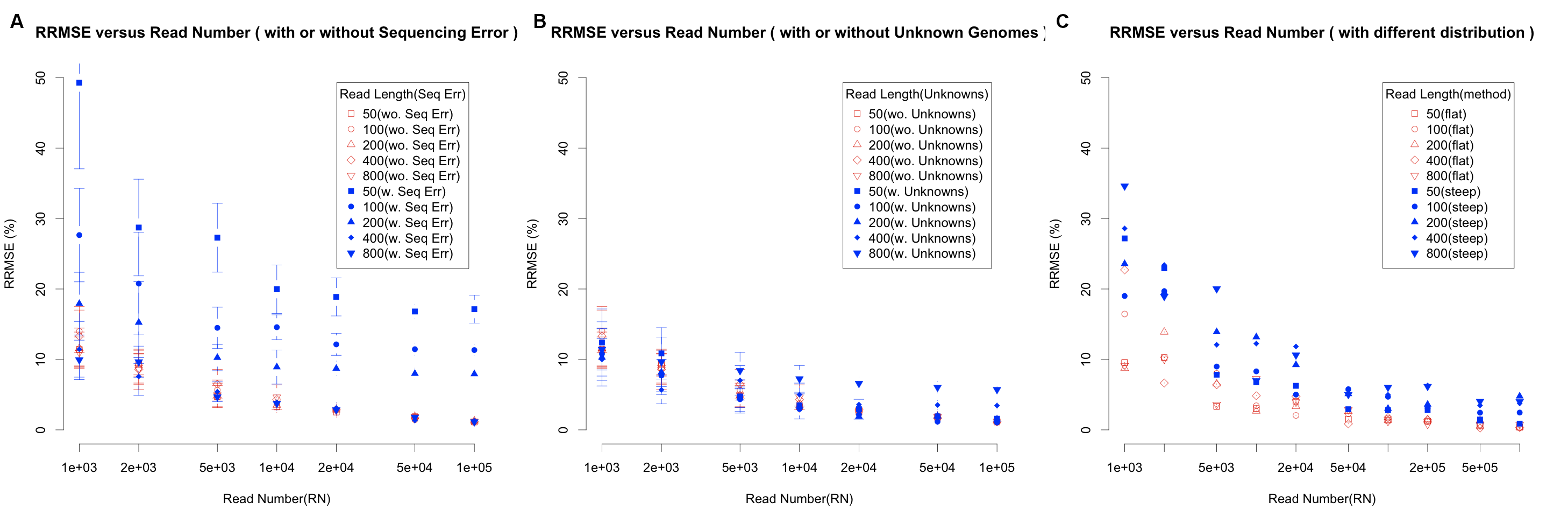

Supplement: Figure S2 — Simulated read set benchmarks. Effects of different perturbations on GRAMMy's estimation: (A) Effects of sequencing errors: results from ‘with sequencing error’ and ‘without sequencing error’ read sets are labeled as ‘w. Seq Err’ and ‘wo. Seq Err’, respectively. (B) Effects of unknown genomes: results from estimation ‘with unknown genomes’ and ‘without unknown genomes’ read sets are labeled as ‘w. Unknowns’ and ‘wo. Unknowns’, respectively. (C) Effects of different genome relative abundance distributions: results from more concentrated abundance distribution and less concentrated read sets are labeled as ‘steep’ and ‘flat’, respectively. Relative Root Mean Square Error (RRMSE) as a percentage is plotted against Read Number. GRAMMy (‘map’) was used. (TIFF) [file pone.0027992.s002.tif]

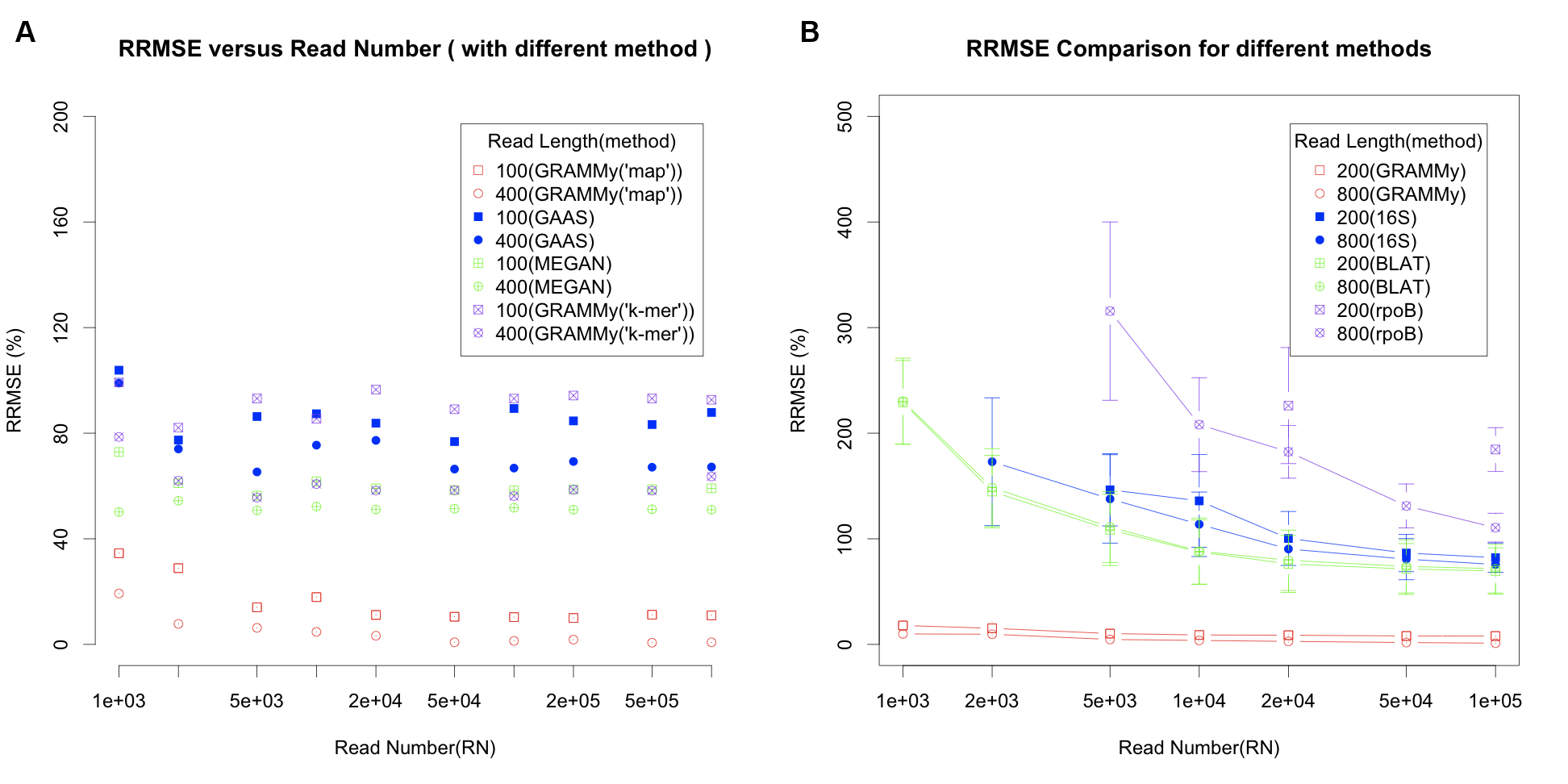

Supplement: Figure S3 — Performance comparison of different methods. The performance comparisons for different estimation methods: (A) MEGAN-based (‘MEGAN’), GAAS (‘GAAS’) and GRAMMy (‘map’ and ‘k-mer’) on simulated read sets with sequencing errors at read length 100 bp and 400 bp. (B) 16S-based (‘16S’), BLAT hit counting (‘BLAT’), rpoB-based and GRAMMy (‘map’). Relative Root Mean Square Error (RRMSE) as a percentage is plotted against Read Number (RN). (TIFF) [file pone.0027992.s003.tif]

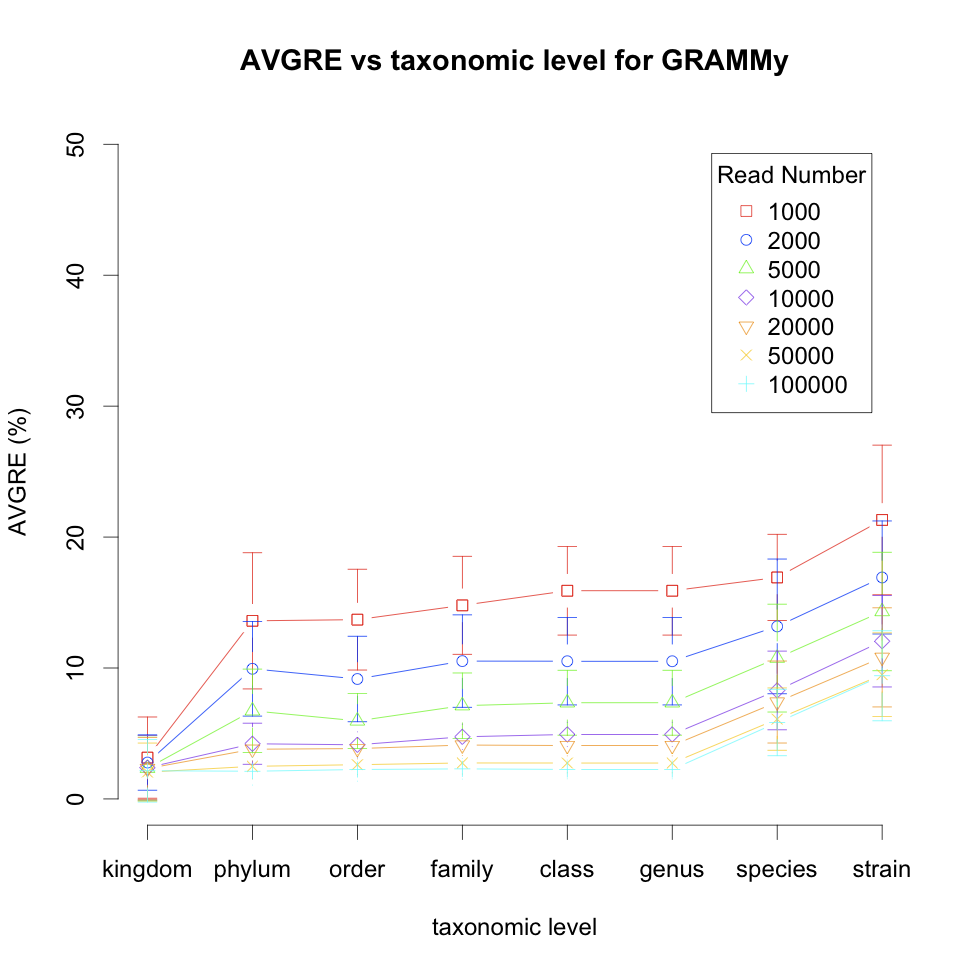

Supplement: Figure S4 — Estimation errors at different taxonomic levels. Average Relative Error (AVGRE) as a percentage is plotted against taxonomic level. The errors gradually decrease from strains to kingdom taxonomic levels. (TIFF) [file pone.0027992.s004.tif]

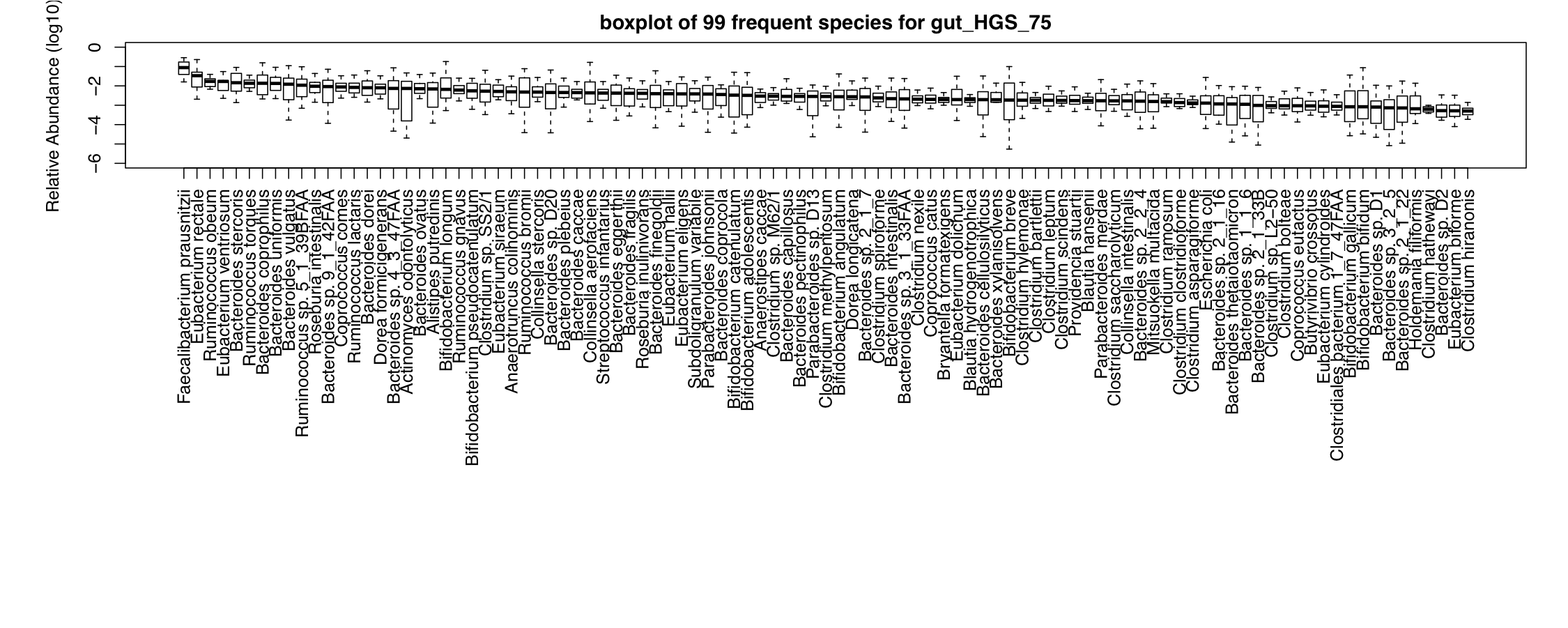

Supplement: Figure S5 — Frequent species for the human gut metagenomes. The 99 species occurring in at least 50% of the 33 human gut samples with a minimum relative abundance of 0.05% were selected. ‘gut_HGS_75’ indicates that the human gut (‘gut’) read sets were mapped to the reference genome set (‘HGS’) with an identity rate cut-off at 75% (‘75’). (TIFF) [file pone.0027992.s005.tif]

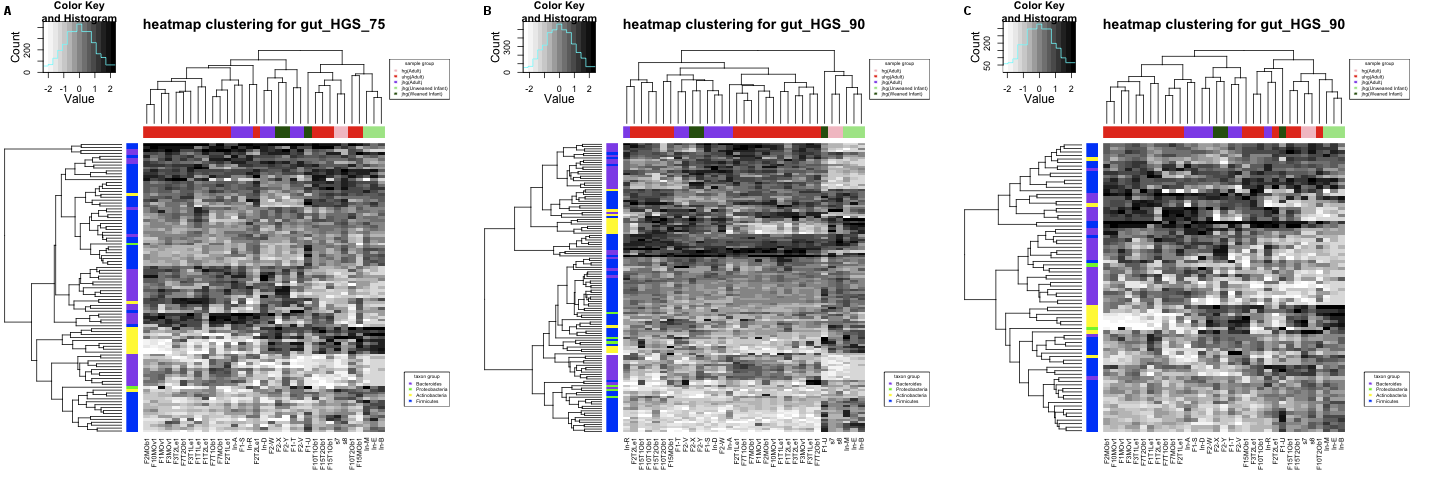

Supplement: Figure S6 — Heatmap biclustering of the human gut metagenomes. ‘gut_HGS_90’ indicates that the human gut (‘gut’) read sets were mapped to the reference genome set (‘HGS’) with an identity rate cut-off at 90% (‘90’), while ‘gut_HGS_75’ indicates cut-off at 75%(‘75’). The bottom labels indicate human gut samples. The top right legend shows the color-coding for columns indicating the sample age category and dataset origin. The bottom right legend shows color-coding for rows indicating the top 4 most abundant phyla in human gut. (A) Heatmap clustering of the ‘gut’ samples, with strains of abundance >0.05% in at least 50% of samples selected at 75% identity rate cut-off. (B) Heatmap clustering of the ‘gut’ samples, with strains of abundance >0.01% in at least 50% of samples selected at 90% identity rate cut-off. (C) Heatmap clustering of the ‘gut’ samples, with strains of abundance >0.1% in at least 50% of samples selected at 90% identity rate cut-off. (TIFF) [file pone.0027992.s006.tif]
