# Supplementary material for: Accurate Genome Relative Abundance Estimation Based on Shotgun Metagenomic Reads
Source: PLoS One. 2011 Dec 6;6(12):e27992. doi: 10.1371/journal.pone.0027992 (PMC3232206; doi:10.1371/journal.pone.0027992)
Supplement: Text S1 — Simulation details and technical derivations. (PDF) [file pone.0027992.s011.pdf]

Supporting Text

Simulated read sets

To evaluate the performance of the GRAMMy, we generated a series of simulated read sets using MetaSim [1], which is a tool specialized to simulate large shotgun metagenomic read sets from input reference genomes and has full-fledged simulating options, such as sequencing error models, population variations and read length distributions.

In our simulation study, we randomly chose ten microbial genomes from the collection of genomes given by the FAMeS study [2]. We then generated an artificial GRA vector from the power-law (*Zipf's*) distribution [3]: $f(k;\alpha,N) = \frac{1/k^\alpha}{\sum_{n=1}^{N} 1/n^\alpha}$ with $\alpha = 2$. Both the reference genomes and the vector of relative abundance were provided to MetaSim, with its population sampling option on, to generate a series of read sets, with RLs (read length) in {50, 100, 200, 400, 800} bp, RN (read number) in {1000, 2000, 5000, 10000, 20000, 50000, 100000}, and SE (sequencing error mode) in either 'with' and 'without sequencing errors'. For each parameter triplet (RL, RN, SE), we generated ten replicates.

To simulate the 'with sequencing errors' scenario, the sequencing errors were introduced into read sets by enabling the '454' or 'Sanger' error mode option in MetaSim to mimic the reads generating behavior of the Roche/454 (RLs = 50-400 bp) and Sanger platforms (RL = 800bp). The read length distribution option was also on to generate reads with normally distributed lengths for the two platforms. These options were conservative because MetaSim was originally published in 2008 and the technologies have been greatly improved since then.

While generating the read replicates, we also permuted the order of all the components in the GRA vector so that every genome had the chance to become either a major or a minor member in the read sets. This permutation procedure reduces the artifact introduced by manually choosing genomes and their abundance levels, as a consequence, the robustness of estimation could be assessed by measuring the standard deviations of all replicates' estimates.

The series of replicated simulated read sets obtained above was then extended with additional non-replicated read sets with RN in {200000, 500000, 1000000} and RL, SE the same as above for larger scale benchmarks.

To evaluate the estimation with different community structures, we randomly generated another GRA vector from the same power law distribution with larger variations in component abundances. We then repeated the above read generation process for all parameter triplets (RL, RN, SE) using this new GRA vector without replicates. This produced a new independent series of read sets with significant differences in microbial community structure from the previous one. We labeled the new series 'steep' since its GRA only had a few dominant species and the previous one 'flat' since its GRA

was more evenly distributed.

Performance evaluation for simulations

We first used the same set of genomes used in read generation as our reference genomes. The alignment program BLAT was used to align the reads to the references and the output was fed into GRAMMy, GAAS and MEGAN. Then, we used different numerical error measures (see "Materials and Methods" in the main text) and their standard deviations to assess the quality of the estimations.

In Figure S1, we plotted the measured errors (with deviation bars) against the read number (RN) to show the convergence of the GRA estimates to their true values. It can be seen from Figure S1A that, as RN increases, the Relative Root Mean Square Error (RRMSE) diminishes to almost zero with decreased variation for all RLs, which indicates, regardless of read lengths, the GRAMMy ('map') accurately converge to their true values and become stable once the read number ensures a high coverage. For instance, when 10^5 reads are available, the RRMSE is less than 2% and its standard deviation is marginal for all RLs.

In Figure S1B, in addition to RRMSE, we measured the Average Relative Error (AVGRE), the Maximum Relative Error (MAXRE), the Distance of Total Variation (DTV) and their standard deviations for the read sets with a RL equal to 100 bp. According to the plot, all four measures converge to zero and stabilize. This pattern is similar using other read lengths. From Figures S1A and S1B, we concluded that the GRAMMy estimation is accurate and robust for different read lengths and error measures.

To further study the performance of GRAMMy within the limitations of partially available reference genomes and current sequencing technologies, we next added more perturbations to the simulation study, such as sequencing errors, unknown genomes. We also applied a different abundance distribution to evaluate the effects from the complexity of a community. The results from these studies were summarized in a series of RRMSE-versus-RN plots in Figure S2.

As we can see from Figure S2A, sequencing errors do affect the estimation accuracy for short reads since the estimation accuracy for read sets 'with sequencing errors' is lower than that for 'without sequencing errors', particularly at RLs $\leq$200 bp. However, for a reasonably large number of reads, a scale routinely achieved in recent metagenomic read sets, the estimates are close to the true values, as in the worst case here, the limiting RRMSE is about 20% for the shortest read length (RL = 50 bp). We can also infer from the plot that, developments from sequencing technologies, such as increased read length and reduced error rates, can help to improve the estimates. For example, at RN equal to 10^5 and 'with sequencing errors', when the RL is increased from 50 to 200 bp, it helps to reduce the

RRMSEs from 20% to 10% approximately. Moreover, when sequencing errors are negligible, 50 bp reads are as informative as any longer ones in the purpose of abundance estimation using our framework.

In reality, inaccuracies in the GRA estimation can also arise from the limited knowledge of reference genomes. In the next simulation, we masked out 50% of the reference genomes and repeated the estimations. As Figure S2B indicates, a partial reference genome set does not substantially affect the accuracy of estimates, despite that they become less robust at a low sequencing depth. In fact, at a sufficient high coverage (RN equal to 10^6), the estimates for read sets 'with unknowns' also converge and is comparably accurate to that of 'without unknowns'. Even if 80% of the reference genomes were masked out, the estimation still had good convergence, as our study indicates (data not shown).

Another factor that may affect the estimation is the community's natural complexity. To study this, we prepared two communities which are different from each other in their shape of GRA distribution. In these read sets, the GRA of the 'flat' sets is more spread among all genomes while that of the 'steep' sets is more concentrated on a few genomes. From the estimations, as shown in Figure S2C, we do not observe significant effects resulting from different complexities, though there are some decrease in accuracy for the 'steep' sets, which may be related to a less coverage of minority genomes.

We also compared GRAMMy to other methods. With the objective of estimating the GRA of communities, we first benchmarked GRAMMy with GAAS. In addition, we included MEGAN, which produces a read profile that summarizes the number of reads assigned to their lowest common ancestors (LCA). We estimated the GRA based on MEGAN using the normalized percentages from the reads distributed on leaf taxon. The default options of GAAS and MEGAN were used in our study. Figure S3A shows the results from the simulation read sets with read lengths (RLs) equal to 100 or 400 bp generated from MetaSim using the with sequencing errors option. We see that GRAMMy ('map') significantly outperformed GAAS, MEGAN and GRAMMy ('k-mer') in all settings. Among all the methods tested, GRAMMy ('map') is the only method with RRMSEs decreasing to zero as the number of reads increases.

In addition to the above methods, We compared the 16S-based, *rpo*B-based and BLAT hit counting estimates to GRAMMy estimates using our simulated read set. Figure S3B shows that GRAMMy outperformed all other methods in this controlled setting. All other methods show three obvious drawbacks: a persisting bias, significant variation and a strong dependence on the number of reads.

Finally, we evaluated the computation time and the error propagation to higher taxonomic levels using our simulated data set. The time and space complexity of our algorithm are shown to be $O(c_1 c_2 n)$ and $O(c_1 n)$, respectively, where n is the size of the read set, c_1 (related to associated

genomes each read) and c_2 (related to EM convergence criteria) are two constants.

We benchmarked GRAMMy with MEGAN and GAAS for running time with different RLs and RNs, see Figure 6. The mapping or alignment time is excluded for all compared tools. We see GRAMMy is consistently faster than the other two in processing the same read set and it scales as expected. In addition, as shown in Figure S4, the errors gradually reduce from lower to higher taxonomic levels. And the error is consistently small when the RN is large. All the simulations are carried out on our “Dell, PE1950, Xeon E5420, 2.5GHz, 12010MB RAM” computing nodes.

In conclusion, our simulations showed GRAMMy estimates are accurate and stable across a range of anticipated settings. Furthermore, it is superior in speed as compared to other available tools. An interesting observation is, when the purpose is to estimate the abundance of a predefined set of reference genomes, an excessively ‘deep sequencing’ scheme is not necessary. As shown in the subfigures of Figure S1-3, the RRMSEs start to stabilize when the RN passes over 10^4 reads, which indicates there may be a threshold for read number that is needed to recover the community abundance structure. This trend also represents that, when the reads ambiguity are properly handled, a read set of relatively smaller number can still provide substantial information for the abundance estimation. Even though the specific threshold value may differ in real settings, it can be predicted using pre-study simulations and is informative for a more economical design of the actual sequencing depth.

Derivation of the EM algorithm

Many estimation methods have been developed for estimating components' mixing parameters for finite mixture models, among which are the Expectation Maximization (EM) algorithm based approaches [4]. The EM based solutions have been proved to be accurate and robust in many cases. Many acceleration methods, like Aitken's, Quasi-Newton and Conjugated Gradient, exist to improve its convergence rate for large size problems. Thus, we adopted the EM based estimation as our solution to the MLE estimation in the transformed mixture problem. In the EM framework, we further assume a ‘missing’ data matrix $\mathbf{Z}$, in which each entry z_{ij} is a random variable indicating whether the read r_i is from the genome g_j. The model then can be solved by estimating π and $\mathbf{Z}$ iteratively using Algorithm 1.

Algorithm 1 Genome Relative Abundance estimation by Finite Mixture Model(GRAMMy)

Require: read set $\mathbf{R}$, reference genomes $\mathbf{G}$, genome lengths $\mathbf{L}$ as inputs.
Variables: missing indices $\mathbf{Z}$, reads probability $\mathbf{f}$, mixing parameters π.
if backend is 'map' **then**
estimate $\mathbf{f}$ by mapping procedures by Equation (5).
end if
if backend is 'k-mer' **then**
estimate $\mathbf{f}$ by k-mer compositions by Equation (6).
end if
Mixing parameters $\pi \Leftarrow Initialize()$ by moment estimates.
repeat
$\pi' \Leftarrow \pi$
E-step: $\mathbf{Z} \Leftarrow Prob(\mathbf{Z}|\pi, \mathbf{R}, \mathbf{G})$ as in Equation (3).
M-step: $\pi \Leftarrow MLE(\pi|\mathbf{Z}, \mathbf{R}, \mathbf{G})$ as in Equation (4).
until π', π converged
Convert $(\pi_1, \pi_2, ..., \pi_{m-1})$ to relative abundance $\mathbf{a}$ by Equation (1).
return a

We will describe the details of the algorithm in the following subsections. Note: a variable with a superscript (t) stands for its value at the t-th iteration in EM, *e.g.* $\pi^{(t)}$ is the estimate of π at the t-th step. The t-th iteration in EM is:

- **E-step**
  Assuming that mixing parameters $\pi^{(t)}$ are known, the 'missing' indicator entries in $\mathbf{Z}^{(t)}$ can be updated using their corresponding posterior probabilities or:

$$\begin{aligned} z_{ij}^{(t)} &= p(z_{ij} = 1|r_i; \pi^{(t)}, \mathbf{G}) \\ &= \frac{p(z_{ij} = 1, r_i|\pi^{(t)}, \mathbf{G})}{p(r_i|\pi^{(t)}, \mathbf{G})} \\ &= \frac{p(r_i|z_{ij} = 1; \pi^{(t)}, \mathbf{G})p(z_{ij} = 1|\pi^{(t)}, \mathbf{G})}{\sum_{k=1}^{m} p(r_i|z_{ik} = 1; \mathbf{a}^{(t)}, \mathbf{G})p(z_{ik} = 1|\pi^{(t)}, \mathbf{G})} \\ &= \frac{p(r_i|z_{ij} = 1; \mathbf{G})\pi_j^{(t)}}{\sum_{k=1}^{m} p(r_i|z_{ik} = 1; \mathbf{G})\pi_k^{(t)}}. \end{aligned} \tag{3}$$

  Notice that we used $p(r_i|z_{ij} = 1; \mathbf{G}, \pi^{(t)}) = p(r_i|z_{ij} = 1; \mathbf{G})$ because of the independence of the two sampling steps in our mixture model and that the read probability $p(r_i|z_{ij} = 1; \mathbf{G})$ can be accessed from $f_{g_j}(r_i|\mathbf{G})$, which is to be approximated using different methods later.

Obviously, the update of $\mathbf{Z}^{(t)}$ depends solely on the updating value of $\pi^{(t)}$.

- **M-step**:
  Now, assuming 'missing' data $\mathbf{Z}^{(t)}$ are known, we calculate new mixing parameters $\pi^{(t+1)}$ that maximize the conditional expectation of the full data log likelihood function $Q(\pi|\pi^{(t)})$ of both the 'missing' and the known data, *i.e.*, we update them using:

$$\pi^{(t+1)} = \arg\ \max_{\pi}\ Q(\pi|\pi^{(t)}),$$

where

$$\begin{aligned} Q(\pi|\pi^{(\mathbf{t})}) &= E(\log\ L(\mathbf{R},\mathbf{Z}|\pi,\mathbf{G})|\mathbf{R},\pi^{(t)}) \\ &= E(\log \prod_{i=1}^{n}\prod_{j=1}^{m}(p(z_{ij}=1|\pi,\mathbf{G})p(r_i|z_{ij}=1;\pi,\mathbf{G}))^{z_{ij}}|\mathbf{R},\pi^{(t)}) \\ &= E(\sum_{i=1}^{n}\sum_{j=1}^{m} z_{ij}(\log\ p(z_{ij}=1|\pi,\mathbf{G}) + \log\ p(r_i|z_{ij}=1;\mathbf{G}))|\mathbf{R},\pi^{(t)}) \\ &= \sum_{i=1}^{n}\sum_{j=1}^{m} p(z_{ij}=1|\pi^{(t)},\mathbf{G})(\log\ p(z_{ij}=1|\pi,\mathbf{G}) + \log\ p(r_i|z_{ij}=1;\mathbf{G})) \\ &= \sum_{i=1}^{n}\sum_{j=1}^{m} \pi_j^{(t)}(\log \pi_j + \log\ p(r_i|z_{ij}=1;\mathbf{G})). \end{aligned}$$

and

$$\log\ L(\mathbf{R},\mathbf{Z}|\pi,\mathbf{G}) = \sum_{i=1}^{n}\sum_{j=1}^{m} z_{ij}(\log\ p(z_{ij}=1|\pi,\mathbf{G}) + \log\ p(r_i|z_{ij}=1;\mathbf{G}))$$

is the model log likelihood function for the complete data ($\mathbf{Z}$, $\mathbf{R}$). The exact form of the maximum likelihood estimator (MLE) for $Q(\pi|\pi^{(\mathbf{t})})$ can be found, and it can be expressed using a simple closed form in $\pi^{(t+1)}$:

$$\pi_j^{(t+1)} = \frac{\sum\limits_{i=1}^{n} z_{ij}^{(t)}}{n}. \tag{4}$$

When the MLE of π is found, using the one-to-one relation in Equation (1), the MLE of $\mathbf{a}$ can be also found, thus we can solve the original biological problem.

Derivation of the standard errors

Using the asymptotic theory for MLE estimates, we can derive the asymptotic covariance matrix for the mixing parameters π. Remember, there are

$m-1$ independent parameters in π we are estimating and let us choose these to be $(\pi_1, \pi_2, ..., \pi_{m-1})$ and denoted by $\hat{\pi}$. Let $\hat{\pi}^*$ and $\mathbf{a}^*$ be the MLE estimates for $\hat{\pi}$ and its corresponding GRA vector. We can derive the observed information matrix $\mathbf{I}_o$,

$$\mathbf{I}_o(\hat{\pi}|\mathbf{R}, \mathbf{G}) = -\frac{\partial^2 \log\ L(\mathbf{R}|\hat{\pi}, \mathbf{G})}{\partial\hat{\pi}\partial\hat{\pi}^T},$$

where:

$$L(\mathbf{R}|\hat{\pi}, \mathbf{G}) = \sum_{i=1}^{n} \log(\sum_{j=1}^{m-1} \pi_j f_{g_j}(r_i|\mathbf{G}) + (1 - \sum_{j=1}^{m-1} \pi_j) f_{g_m}(r_i|\mathbf{G}))$$

is the log likelihood function of the observed data $\mathbf{R}$. Therefore, we write each entry of $\mathbf{I}_o$ as:

$$\mathbf{I}_o(\hat{\pi}|\mathbf{R}, \mathbf{G})_{kl} = \sum_{i=1}^{n} \frac{(f_{g_k}(r_i|\mathbf{G}) - f_{g_m}(r_i|\mathbf{G}))(f_{g_l}(r_i|\mathbf{G}) - f_{g_m}(r_i|\mathbf{G}))}{(\sum_{j=1}^{m-1} \pi_j f_{g_j}(r_i|\mathbf{G}) + (1 - \sum_{j=1}^{m-1} \pi_j) f_{g_m}(r_i|\mathbf{G}))^2},$$

for $k, l \in \{1, 2, ..., m-1\}$. Because the GRA vector $\mathbf{a}$ is a rank preserving transformation of $\hat{\pi}$, we can subsequently write the observed information matrix $\mathbf{I_o}(\mathbf{a}|\mathbf{R}, \mathbf{G})$ with regard to the parameterization of $\mathbf{a}$ as:

$$\mathbf{I_o}(\mathbf{a}|\mathbf{R}, \mathbf{G}) = \nabla_{\mathbf{a}}(\hat{\pi})^T \mathbf{I}_o(\hat{\pi}|\mathbf{R}, \mathbf{G}) \nabla_{\mathbf{a}}(\hat{\pi}),$$

and the asymptotic standard error for our MLE estimate a_j^* as:

$$SE(a_j^*) = (\mathbf{Cov}(\mathbf{a}^*))_{jj} \approx ((\mathbf{I_o^{-1}}(\mathbf{a}|\mathbf{R}, \mathbf{G}))_{jj})^{\frac{1}{2}}|_{\hat{\pi}=\hat{\pi}^*}, \quad (7)$$

for $j \in \{1, 2, ..., m-1\}$, considering $\hat{\pi}$ as the natural parameter set and $\mathbf{a}$ as another parameter set, and that the asymptotic variance matrix can be effectively calculated by taking the inverse of the observed information matrix [5] and the standard error is the square root of variance entries on the diagonal. Finally, we use Equation (7) as our standard errors for our GRA estimates.

However, when the number of reads as compared to number of parameters is small or the majority of reads fails to be mapped, the asymptotic condition is not met and the application of previous result is not valid. However, we can still use the bootstrap estimator for covariance to estimate the standard error of our MLE using the empirical distribution:

$$SE(a_j^*) = (\mathbf{Cov}(\mathbf{a}^*))_{jj} \approx \left(\frac{1}{B-1}\sum_{b=1}^{B}(\mathbf{a}_{(b)}^* - \bar{\mathbf{a}}^*)(\mathbf{a}_{(b)}^* - \bar{\mathbf{a}}^*)^T\right)_{jj}, \quad (8)$$

where $\bar{\mathbf{a}}^* = \frac{1}{B}\sum_{b=1}^{B} \mathbf{a}_{(b)}^*$ is the bootstrap mean estimator of the samples' MLEs.

Convergence of the EM algorithm

Because the EM method is greedy, it may not converge to the global maximum of the objective function. However, in this case, we shall show the observed data log likelihood function $L(\mathbf{R}|\hat{\pi}, \mathbf{G})$ is concave with regard to $\hat{\pi}$. Thus, any local maximum the EM converge to is the global maximum.

PROPOSITION 1. $L(\mathbf{R}|\hat{\pi}, \mathbf{G})$ is concave.

PROOF. Since the sum of concave functions is still concave, proving the concavity of the log likelihood function of single observation suffices. Taking the second-order derivatives of the summands of $L(\mathbf{R}|\hat{\pi}, \mathbf{G})$, we have

$$\frac{\partial^2 \log\ L(r_i|\hat{\pi}, \mathbf{G})}{\partial \hat{\pi} \partial \hat{\pi}^T} = -\frac{(f_{g_k}(r_i|\mathbf{G}) - f_{g_m}(r_i|\mathbf{G}))(f_{g_l}(r_i|\mathbf{G}) - f_{g_m}(r_i|\mathbf{G}))}{(\sum_{j=1}^{m-1} \pi_j f_{g_j}(r_i|\mathbf{G}) + (1 - \sum_{j=1}^{m-1} \pi_j) f_{g_m}(r_i|\mathbf{G}))^2} \quad (9)$$

If consider the Hessian matrix $\mathbf{H}$ where the (k, l)-th element is Equation (9), we can write $\mathbf{H}$ as $\mathbf{H} = -d\mathbf{v}^t\mathbf{v}$, where

$$\mathbf{v} = (f_{g_1}(r_i|\mathbf{G}) - f_{g_m}(r_i|\mathbf{G}), ..., f_{g_{m-1}}(r_i|\mathbf{G}) - f_{g_m}(r_i|\mathbf{G}))$$

is a vector and

$$d = \frac{1}{(\sum_{j=1}^{m-1} \pi_j f_{g_j(r_i|\mathbf{G})} + (1 - \sum_{j=1}^{m-1} \pi_j) f_{g_m}(r_i|\mathbf{G}))^2}$$

is a scalar. Notice $d \geq 0$, therefore $\mathbf{H}$ is negative semi-definite because for any vector $\mathbf{u} = (u_1, ..., u_{m-1})$, we have $\mathbf{u}\mathbf{H}\mathbf{u}^t = -d(\mathbf{u}\mathbf{v}^t)(\mathbf{u}\mathbf{v}^t)^t = -d(\mathbf{u}\mathbf{v}^t)^2 \leq 0$. Thus, the concavity of the log likelihood function $L(\mathbf{R}|\hat{\pi}, \mathbf{G})$ is proved.

References

[1] Richter DC, Ott F, Auch AF, Schmid R, Huson DH (2008) MetaSim: a sequencing simulator for genomics and metagenomics. PLoS ONE 3: e3373.

[2] Mavromatis K, Ivanova N, Barry K, Shapiro H, Goltsman E, et al. (2007) Use of simulated data sets to evaluate the fidelity of metagenomic processing methods. Nat Methods 4: 495–500.

[3] Marquet PA, Quiones RA, Abades S, Labra F, Tognelli M, et al. (2005) Scaling and power-laws in ecological systems. J Exp Biol 208: 1749–1769.

[4] Dempster A, Laird N, Rubin D, et al. (1977) Maximum likelihood from incomplete data via the EM algorithm. Journal of the Royal Statistical Society Series B (Methodological) 39: 1–38.

[5] Efron B, Hinkley D (1978) Assessing the accuracy of the maximum likelihood estimator: Observed versus expected Fisher information. Biometrika 65: 457.
